# Mountains too high and valleys too deep drive population structuring and demographics in a Qinghai–Tibetan Plateau frog *Nanorana pleskei* (Dicroglossidae)

**DOI:** 10.1002/ece3.2646

**Published:** 2016-12-18

**Authors:** Weiwei Zhou, Jieqiong Jin, Jun Wu, Hongman Chen, Junxiao Yang, Robert W. Murphy, Jing Che

**Affiliations:** ^1^ State Key Laboratory of Genetic Resources and EvolutionKunming Institute of ZoologyChinese Academy of SciencesKunmingChina; ^2^ Nanjing Institute of Environmental SciencesMinistry of Environmental ProtectionNanjingChina; ^3^ Kunming College of Life ScienceUniversity of Chinese Academy of SciencesKunmingChina; ^4^ Centre for Biodiversity and Conservation BiologyRoyal Ontario MuseumTorontoONCanada

**Keywords:** amphibians, demographic history, eastern Qinghai–Tibetan plateau, Pleistocene climatic oscillations, population structure

## Abstract

Pleistocene glacial–interglacial climatic oscillations greatly shaped the current genetic structure of many species. However, geographic features may influence the impact of climatic cycling. Distinct geographic and environmental characters between northern and southern parts of the eastern Qinghai–Tibetan Plateau (EQTP) facilitate explorations into the impacts of geographic features on species. The northern parts of EQTP contain large areas of marsh, and the environment is rather homogeneous. In contrast, the southern EQTP harbors complex alpine valleys and a much more heterogeneous setting. We evaluate DNA sequence variation from both the mitochondrial and nuclear genomes in *Nanorana pleskei*, a species endemic to the EQTP. Hypothesis testing on the evolutionary history of *N. pleskei* indicates that northern populations can disperse freely, but alpine valleys isolate southern populations. Demographic histories between northern and southern populations also differ. Northern populations appear to have experienced population expansions, while southern frogs exhibit a far more stable demographic history. By combining climatic analyses and species' distribution models, our study suggests that geographic and environmental features drive the differences between the northern and southern EQTP.

## 1 Introduction

Pleistocene climatic cycling is one of the most important drivers of contemporary diversity in many temperate species and communities. Following the global cyclical cooling–warming events in the Quaternary, the distributions of various organisms experienced concomitant expansions and contractions. During this process, vicariance drove the divergence of populations, and the disappearance of barriers resulted in dispersal and secondary contact. These cyclical events appear to have shaped the current genetic structure of most temperate species (Hewitt, 2000, 2004). Notwithstanding, geographic features also influence the distributions of species, especially in regions not covered by a unified ice sheet. Geographic barriers may promote vicariance in association with climatic changes. For example, barriers may stop dispersal and, thus, restrict expansion. Complex geographic features may harbor suitable microhabitats in the form of micro‐refugia. These refugia may buffer the impacts of climate changes and shape patterns of genetic diversity by stabilizing populations. In contrast, fluctuations in population size of organisms are easy to happen in the homogeneous environment.

**Figure 1:**
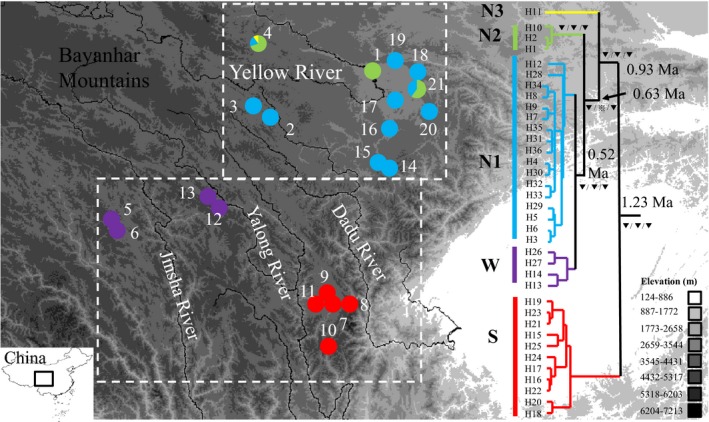
Sampling sites and the maternal genealogy of *Nanorana pleskei* based on trees yield from beast. Site numbers detailed in Table S1. Colors of lineage correspond with localities on the map. Bootstrap proportions (maximum‐likelihood [ML] and maximum parsimony [MP] trees) ≥70% and Bayesian posterior probabilities ≥95% were treated as strong support (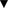). Bootstrap proportions ≥50% and Bayesian posterior probabilities ≥75% were treated as weakly support (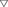). Bootstrap proportions <50% and Bayesian posterior probabilities <75% were treated as not support (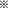). Support rates of the nodes display following Bayesian inference/ML/MP. Numbers in nodes indicate divergence times yield from beast. The two dash‐line boxes indicate nEQTP and sEQTP. The black lines indicate rivers

The complex geographic features and dramatic changes in climate make the eastern edge of the Qinghai–Tibetan Plateau (EQTP) an ideal place for exploring the impacts of the environment on organisms. The EQTP consists of distinctive northern and southern parts based on their differing environments. The northern EQTP (nEQTP) occurs east of the Bayanhar Mountains and north of the Qionglai Mountains (Figures S1 and 1). It largely consists of high‐elevation plains, and the environment is more homogeneous than in the southern EQTP (sEQTP). The nEQTP has a few rolling hills void of steep slopes. The Yellow River flows through it (Figures 1 and S1). The upstream Dadu River contains two major branches—the Maerke and Duoke rivers—that flow from north to south. Large areas of marsh form along these rivers.

The sEQTP exists south of the Bayanhar Mountains (You & Yang, 2013). This region belongs to the Hengduan Mountains, which are famous for their complex environmental features and diverse ecozones. These features contribute to it being a biodiversity hot spot (Myers, Mittermeier, Mittermeier, Da Fonseca, & Kent, 2000). The sEQTP consists of heterogeneous alpine valleys made by four longitudinal mountains separated by several major branches of the Yangtze River. From east to west, the Dadu River flows between the Qionglai and Daxueshan mountains, the Yalong River flows between the Daxueshan and Shaluli mountains, and the Jinsha River flows west of Shaluli Mountain (Figures S1 and 1). These rivers form deeply incised valleys. The environment, including temperature, precipitation, and vegetation forms, changes drastically in the valley. The environment is arid and hot at bottom of these valleys, especially in the middle of Hengduan Mountains which range from 28°N to 32°N (You & Yang, 2013). The environments in the valleys potentially form barriers to dispersal. Drastic environmental differences between sEQTP and its adjacent region, nEQTP, provide opportunities to test how the environment influences genetic diversity and further understand the factors driving the regional biodiversity forming.

Although much research focuses on species in this region, very few studies evaluate the effects of rivers as geographic barriers on organisms. Among the exceptions, *Scutiger boulengeri* is an Alpine stream frog of the sEQTP, and its matrilineal genealogy suggests that the Yalong and Dadu rivers are effective barriers (Li, Chen, Tu, & Fu, 2009). In the lesser striped shrew (*Sorex bedfordiae*), divergences of the matrilines roughly correspond to large rivers (Chen et al., 2015). In contrast, rivers alone failed to explain the population structure of *Rana kukunoris* (Zhao, Dai, & Fu, 2009) and a range‐wide study of this species (Zhou et al., 2013), which did not test specifically for river effects, also failed to discover that rivers served as barriers to dispersal.

The EQTP experienced drastic climatic changes during the Quaternary. Although no unified ice sheet formed, isolated montane glaciers, ice caps, and valley glaciers existed, especially in the sEQTP (Zhou, Wang, Wang, & Xu, 2006). Pollen records suggest that permafrost/desert steppe largely covered the QTP during the Last Glacial Maximum (LGM), yet forest islands existed in the southeastern and eastern ranges (Tang & Shen, 1996; Tang, Shen, Kong, Wang, & Liu, 1998). Glacial refugia, which have contributed greatly to the formation of genetic diversity pattern and strongly impacted demographic histories, occur commonly in the sEQTP (Chen et al., 2008; Li et al., 2011; Wang et al., 2011; Yue & Sun, 2014). Refugia in the nEQTP are also reported (Jia, Liu, Wang, Zhou, & Liu, 2011; Zhou et al., 2013). Because geological features may influence demographic history, and given the differences in geography between northern EQTP and southern EQTP, we test hypotheses related to the impact of climatic cycling and geography on organisms.

To reveal the impacts of climatic changes and geographic features on organisms, we use an endemic, medium‐sized frog, *Nanorana pleskei* (Dicroglossidae). It occurs commonly in open habitats of the EQTP at elevations ranging from 3,000 to 4,500 m (Figure S1). The species prefers a lentic environment, including marshes, pools, and ponds (Fei, Hu, Ye, & Huang, 2009). Given its relatively low mobility and narrow physiological requirements on the high‐elevation region, we hypothesize that geographic features limit its dispersal. Consequently, we test the prediction that the species retains abundant signals of historical responses. Our study has the potential to yield insights into how the environment affects the fauna of the EQTP.

Herein, we report on genetic analyses of a collection of samples from across the entire range of *N. pleskei*. We use gene sequences and climatic data to test two primary hypotheses: (1) different geographic features in northern EQTP and southern EQTP influenced *N. pleske*i in a similar way and (2) the different environments in northern EQTP and southern EQTP impacted the demographic history of *N. pleskei* in a similar way.

## 2 Materials and Methods

### 2.1 Sampling and sequencing

We sampled 181 individuals from 21 localities covering most of the distribution (Figure S1) of *N. pleskei* (Figure 1; Table S1). Two samples of *N. parkeri* were included as the out‐group taxa based on previous studies (Che et al., 2010). All collecting adhered to animal‐use protocols approved by the Kunming Institute of Zoology Animal Care and Ethics Committee.

DNA was extracted from tissue samples using the standard phenol–chloroform extraction protocol (Sambrook & Russell, 2001). Partial sequences of mitochondrial cytochrome b (*Cytb*) (Zhou et al., 2012) and cytochrome C oxidase subunit I (*COI*) (Che et al., 2012) were sequenced for all samples. We also amplified and sequenced nuclear DNA (nuDNA) markers for subsets of samples, including recombination activating gene 1 (*RAG‐1*) (Stuart, 2008), tyrosine precursor exon 1 (*Tyr*) (Bossuyt & Milinkovitch, 2000), and partial sequences from an intron of proglucagon (*GCG*) (Yan et al., 2013; Zhou et al., 2012). Primers used for PCR and sequencing are shown in Table S2. Fragments were subjected to purification using EXO‐SAP (Takara Bio Inc., Shiga, Japan). Both forward and reverse directions were sequenced to preclude sequencing errors.

Obtained sequences were aligned using clustalx 1.81 (Thompson, Gibson, Plewniak, Jeanmougin, & Higgins, 1997) with default parameters. Nuclear gene sequences containing more than one ambiguous site were resolved using phase 2.1.1 (Stephens & Scheet, 2005; Stephens, Smith, & Donnelly, 2001), and input files for phase were prepared using seqphase (Flot, 2010). The longest nonrecombining regions for nuclear locus were generated by imgc (Woerner, Cox, & Hammer, 2007). dnasp 5.10 (Librado & Rozas, 2009) was used to generate identical haplotypes for mtDNA haplotypes and alleles of phased nuDNA.

### 2.2 Genealogy reconstruction and population relationship inference

We used Bayesian inference (BI), maximum‐likelihood (ML), and maximum parsimony (MP) to infer the matrilineal genealogy from mtDNA sequences. The best partition strategy and nucleotide substitution model for each data partition were determined using partitionfinder 1.1.1 (Lanfear, Calcott, Ho, & Guindon, 2012). After assigning the corresponding model to each partition, BI analyses were performed using mrbayes 3.1.2 (Ronquist & Huelsenbeck, 2003). Ten million generations of metropolis‐coupled Markov chain Monte Carlo chains with default heating values were used, and the first 50% of sampled trees were discarded as burn‐in. Effective sample sizes (ESS) were checked using tracer 1.6 (Rambaut, Suchard, Xie, & Drummond, 2014), and parameters with ESSs lower than 200 were treated as unreliable. We conducted ML analyses in raxml gui (Silvestro & Michalak, 2012) with 1,000 bootstrap pseudoreplicates. The partition strategy was as same as BI, and the GTRGAMMA model was implemented for each data partition. paup* 4.0b10a (Swofford, 2003) was used to perform MP analyses. All characters were treated as unordered and equally weighted. Heuristic searches with tree bisection reconnection were executed for 1,000 random addition replicates. Nodal reliability was assessed using 1,000 bootstrap pseudoreplicates.

Based on the combined mtDNA and nuDNA data, we estimate relationships among different groups using *beast (Heled & Drummond, 2010). *RAG‐1* was not used as all variations were in heterozygous states. We defined three groups: W, S, and N. Groups W and S were defined based on mtDNA genealogy and distribution. There are three matrilines in nEQTP. Matriline N3 was not included as it was represented by only one individual and we failed to amplify *GCG* of this sample, although we tried several times. We tested whether samples from nEQTP (matrilines N1 and N2) deviated from Hardy–Weinberg equilibrium (HWE) using arlequin 3.5 (Excoffier, Laval, & Schneider, 2005). If we cannot detect significant deviations, N1 and N2 would be combined as one group (N) in the analyses. Sequences from *Quasipaa boulengeri* were obtained from another study (Yan et al., 2013) and used as the out‐group. No shared mtDNA haplotype was found among the three groups. The results of dispersal model test supported that they were completely isolated from each other. Thus, significant violation of the assumption of the method, no gene flow between groups, was not detected in our datasets. A strict molecular clock model was assumed for all loci, and each locus was specified with its own model. We also performed same analyses using nuDNA data only, in case of discordances between mtDNA and nuDNA data. We ran the MCMC chain for 100 million iterations; sampling every 10,000 generation, first 50% trees were discarded as burn‐in. Effective sample sizes were checked using tracer 1.6 (Rambaut et al., 2014) to ensure the convergence. Population trees were visualized using densitree (Bouckaert, 2010).

To visualize overall similarity of the haplotypes, we built a median‐joining network (MJN) using network 4.5.13 (Bandelt, Forster, & Rohl, 1999) based on mitochondrial data. Nuclear data were not involved in this analysis due to a dearth of variation. The MP option was used to remove excessive links and median vectors (Polzin & Daneshmand, 2003).

### 2.3 Divergence times

Strict molecular clock hypothesis was tested using the likelihood ratio test. We used phylip 3.69 (Felsenstein, 2004) to assess the likelihood of unconstrained and clock‐enforced matrilineal genealogies. The chi‐square test was used to test the significance (Felsenstein, 1981). A priori, we decided to apply this model if the strict molecular clock hypothesis could not be rejected (Ho & Duchêne, 2014).

Times of divergence were estimated in beast 1.8.1 (Drummond, Suchard, Xie, & Rambaut, 2012) based on mitochondrial data. The divergence time between *N. pleskei* and *N. parkeri* (8.9 ± 2.7 Ma) (Che et al., 2010) was used as a secondary calibration point. We performed analyses for 50 million generations and sampled trees every 1,000 generations. Burn‐in and convergence of the chains were determined with tracer 1.6. The ESS of each parameter was also checked using tracer 1.6 to confirm acceptable convergence.

### 2.4 Genetic structure and demographic history

Populations of *N. pleskei* were grouped by using spatial analysis of molecular variation (SAMOVA) (Dupanloup, Schneider, & Excoffier, 2002), which was implemented in spads 1.0 (Dellicour & Mardulyn, 2014). The number of groups (*K*), which was allowed to range from 2 to 10, was explored with 10,000 iterations and 100 repetitions. The optimum value of *K* was identified by exploring the behavior of the proportion of total genetic variance due to differences between groups of populations (FCT). We explored multiple *K* values after FCT reached the plateau (Meirmans, 2015). We also explored the spatial pattern of nuDNA alleles to check whether they were concordant with the population grouping based on mtDNA. To visualize the spatial pattern of nuDNA alleles, we mapped allele frequencies on each locality for three loci separately.

We assessed the demographic history based on mtDNA data of each matrilineal lineage separately, because population subdivision may have influenced detection of demographic shifts. Firstly, we calculated Tajima's *D* (Tajima, 1989) and Fu's *F*s statistics (Fu, 1997) using arlequin 3.5 (Excoffier et al., 2005). The significance of each test was assessed by generating null distributions from 10,000 coalescent simulations. Secondly, we tested for signals of demographic expansion by mismatch distributions (Rogers & Harpending, 1992) for each lineage as implemented in arlequin 3.5. The sudden demographic expansion was used as the null model, and the null distribution was obtained by generating 10,000 permutations. The significance of deviations from this model was tested by using the sum of squared deviation and raggedness index (Rag).

### 2.5 Species distribution model building

The maximum entropy model implemented in maxent 3.3.3 (Phillips, Anderson, & Schapire, 2006; Phillips & Dudík, 2008) was used to generate the species distribution model (SDM). Occurrence data were based on GPS data taken during our field work. Bioclimatic variables with resolutions of 30 arc seconds were downloaded from the WorldClim database (Elith et al., 2006; Hijmans, Cameron, Parra, Jones, & Jarvis, 2005; Hijmans & Graham, 2006). All layers were cropped to span from 95°E to 107°E and from 28°N to 40°N. Bioclimatic data for LGM were disaggregated into same resolution as current data. All of the 19 bioclimatic layers were used in analyses because of the controversy about whether correlated variables should be removed or not (Merow, Smith, & Silander, 2013).

We used random null distributions to test whether the SDM of *N. pleskei* was significantly better than the random distribution (Smith, 2013). Twenty‐one random points (the same number as sampling localities) across the study range were used to generate the SDM. We repeated this process 100 times and extracted the values of areas under the curves (AUCs) as the null distribution.

Assuming niche conservatism over time (Holt, 2003; Peterson, Soberón, & Sánchez‐Cordero, 1999; Wiens & Graham, 2005), we projected the SDM to the climatic data layers for the LGM to obtain the predicted distribution. Both the community climate system model (CCSM) (Collins et al., 2006) and the model for interdisciplinary research on climate (MIROC) (Hasumi & Emori, 2004) were used to reconstruct the paleodistribution. A ten‐percentile training presence threshold rule was applied to the training for all analyses. Further, we assessed distributional expansion after the LGM by comparing the number of grids that were suggested as being suitable for *N. pleskei* after applying the threshold. First, we compared the overall changes. Next, because different areas might have displayed different patterns, we divided the study range into northern and southern regions by 32°N. When projecting the SDM into the LGM climate layers based on the MIROC, large areas of suitable habitats were detected in northeastern Tibet, which was far from current and reported distribution of *N. pleskei*. Thus, we compared distribution changes after removing this region.

### 2.6 Testing the barrier effects of rivers

We tested for potential barrier effects of rivers using two approaches. Firstly, we tested the hypothesis that the rivers did not stop dispersal. For the test, we divided populations into six regions according to the four major rivers in the EQTP (Figure S1) and calculated *F*_st_ matrix. Then, we set 17 dispersal models among these groups. Details of each model are summarized in Table 1. Dispersals between populations were estimated by using migrate‐n 3.2.6 (Beerli, 2006; Beerli & Felsenstein, 2001). Model comparison was done using Bayes factor test (BFT) following Beerli's methods (Beerli & Palczewski, 2010). The natural log Bayes factors (LBF) were calculated following the formula: LBF = 2 (ln(Prob(*D* |Model A) ‐ ln(Prob(*D*|Model i)), while Model A stood for the model with largest log likelihood. LBF larger than 2 suggests Model A was significantly better than Modle i. migrate‐n was run with one long MCMC chain for 5 million generations while sampling every 1,000 generations and discarding the first 10,000 genealogies as burn‐in. Static heating was employed, and temperatures of each Markov chain were set as 1, 1.5, 3, and 10 million. Performance of each run was checked by assessing effective sample size.

**Table 1:** Results of dispersal models selection

	Model description	Raw thermodynamic score	LBF	Probability	Bezier approximation score	LBF	Probability
Full	Full model	−3653.805	1512.662	0.00	−2482.805	66.058	0.00
1	No isolation among populations	−3582.769	1370.198	0.00	−2468.876	38.198	0.00
2	Jinsha River is barrier	−3421.088	1046.836	0.00	−2450.449	1.344	0.34
3	Yalong River is barrier	−3106.254	417.168	0.00	−2583.104	266.654	0.00
4	Dadu River is barrier	−3038.147	280.952	0.00	−2584.113	268.674	0.00
5	Yellow River is barrier	−3555.270	1315.200	0.00	−2471.900	44.246	0.00
6	Jinsha and Yalong rivers are barriers	−3253.170	711.000	0.00	−2705.948	512.342	0.00
7	Jinsha and Dadu rivers are barriers	−3124.144	452.946	0.00	−2581.636	263.718	0.00
8	Jinsha and Yellow rivers are barriers	−3505.713	1216.086	0.00	−2603.680	307.806	0.00
9	Yalong and Dadu rivers are barriers	−2911.174	27.008	0.00	−2449.777	0	0.66
10	Yalong and Yellow rivers are barriers	−3207.566	619.790	0.00	−2628.006	356.460	0.00
11	Dadu and Yellow rivers are barriers	−3390.442	985.542	0.00	−2883.358	867.164	0.00
12	Yalong, Dadu and Yellow rivers are barriers	−3358.488	921.634	0.00	−2860.695	821.838	0.00
13	Jinsha, Dadu and Yellow rivers are barriers	−3163.686	532.030	0.00	−2600.010	300.468	0.00
14	Jinsha, Yalong and Yellow rivers are barriers	−3332.023	868.706	0.00	−2820.995	742.438	0.00
15	Jinsha, Yalong and Dadu rivers are barriers	−2897.670	0	1.00	−2482.481	65.408	0.00
16	All rivers are barriers	−3122.420	449.500	0.00	−2644.957	390.362	0.00

LBF, log Bayes factors.

We also tested the hypothesis that climate along rivers was suitable for *N. pleskei*. If true, different rivers should have similar suitability scores as localities for *N. pleskei* in the SDMs and bioclimatic factors of rivers should be concordant with sampling localities. To test the hypothesis, we generated points for all major rivers in this range and then extracted the values of each point from the corresponding layer of the SDM after applying the chosen threshold. We mapped these points to check which parts of the rivers were suitable for *N. pleskei* and which were not. Secondly, we generated points for the four major rivers separately. Subsequently, we extracted the value of probability that conditions were suitable from the SDM layer and then compared these data to the data extracted from sampling localities of *N. pleskei*. As environment along the rivers might be quite different, we used box plots to check whether habitat suitability of rivers was partly overlapping with *N. pleskei*. Finally, we compared environmental differences among the rivers and *N. pleskei*. Values for bioclimatic variables for all sampling sites of *N. pleskei* and points of each river were extracted using diva‐gis (Hijmans et al., 2012). We checked whether the data violated normal distribution using spss 16.0 and the bioclimatic variable which skewness and kurtosis values were larger than one were treated as violated the normal distribution. Then, we transformed these bioclimatic variables into normal distribution using SPSS 16.0. Principal component analysis (PCA) was performed using spss 16.0, and the principal components (PCs) with an eigenvalue larger than one were kept for further analyses. Scatter plots were made from PCs to visualize the environmental similarity of rivers and *N. pleskei*.

## 3 Results

### 3.1 Sequence information

MtDNA markers *Cytb* and *COI* were sequenced for all samples. A total of 576 bp of *Cytb* contained 101 variable and 78 potentially parsimony‐informative (PPI) sites. For *COI*, the 540‐bp fragment had 88 variable and 69 PPI sites after trimming the ends. The concatenated sequences resolved 38 haplotypes, including the out‐group taxa. Sequences for the three nuclear genes were obtained from a subset of our samples (Table S1). *RAG‐1* sequences of 1162 bp were obtained from 55 frogs and these included two variable sites, and *Tyr* fragments of 617 bp from 32 samples had one variable site. Twenty‐eight sequences of GCG were obtained with 817 bp, which included four variable sites.

### 3.2 Matrilineal genealogy and population relationships inference

The best partition strategy of the concatenated mtDNA data was dividing by codon positions. First codon position used the K80 model, second codon position used the F81 model, and third codon position used the HKY + G model. MP, ML, and BI analyses yielded similar topologies for the matrilineal genealogy (Figures 1 and S2). Incongruent nodes received weak supports. For example, in MP tree, Matriline S was recovered as a monophyly but not strongly supported. In BI and ML trees, this monophyly was not recovered.

Five matrilines, S, N1, N2, N3, and W, were defined. Haplotypes H15–H25 from localities 7 to 11 were considered to constitute Matriline S for demographic analyses due to their geographic cohesiveness and co‐occurrences. Matriline S located in the southern region between the Dadu and Yalong rivers constituted five lineages of largely unresolved relationships that rooted at the base of the genealogy for *N. pleskei*. Excluding S, all other samples grouped into a strongly supported, geographically circumscribed lineage that consisted of four matrilines. Within this lineage, Matriline N3 (H11), which was detected in one of 10 individuals from locality 4 only, was highly divergent from all other haplotypes and rooted as the sister group of the remaining lineages. The remaining haplotypes formed three matrilines. Matriline N2 contained H1, H2, and H10 from localities 1, 4, and 21. Matriline N1 contained haplotypes H3–H9, H12, and H28–H36, which were widely distributed in nEQTP. Haplotypes from localities 5, 6, 12, and 13, which lay west of the Yalong River, clustered together and formed Matriline W. Within it, samples from localities 5 and 6 clustered together as did samples from localities 12 and 13. The Jinsha River separated these two sublineages. Matrilines N1 and W clustered together with strong support.

**Figure 2:**
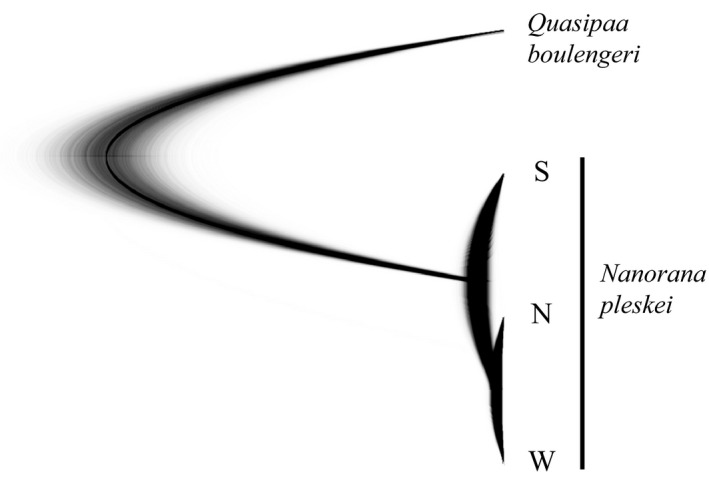
Cloudogram of the population tree analyses based on combined mtDNA and nuDNA data

Significant deviation from Hardy–Weinberg equilibrium (HWE) was not detected in *Tyr* (*p* = .53) and *GCG* (*p* = .33). Samples from nEQTP (matrilines N1 and N2) were combined together. The population tree based on the combined mtDNA and nuDNA data (Figure 2) consisted of mtDNA genealogy (Figure 1 and S2). Population W and N clustered together, and Population S was placed at a basal position. Population tree based on two nuDNA loci yielded the same topology with tree based on combined data, but with much lower posterior probability (Figure S3).

The MJN based on the mtDNA (Figure S4) was congruent with the mtDNA genealogy (Figures 1 and S2). The five major matrilines were depicted. Matriline N1 contained 17 haplotypes and formed a star‐like pattern with most common H3 in the center. Haplotypes of Matriline S clustered together.

### 3.3 Divergence time estimation

A strict molecular clock, the null hypothesis, was not rejected (χ^2^ = 16.60, *df* = 36, *p* = .998). In the trees yielded from beast, monophyly of Matriline S was recovered with strong support (with posterior = 1.0). The radiation of *N. pleskei* happened about 1.23 Ma (95% CI: 0.68–1.68 Ma) during the Pleistocene. Matriline N3 split from N1, N2, and W around 0.93 Ma (95% CI: 0.52–1.27 Ma). Matrilines N1 and W diverged about 0.52 Ma (95% CI: 0.26–0.67 Ma) (Figure 1).

### 3.4 Genetic structure and demographic history

**Figure 3:**
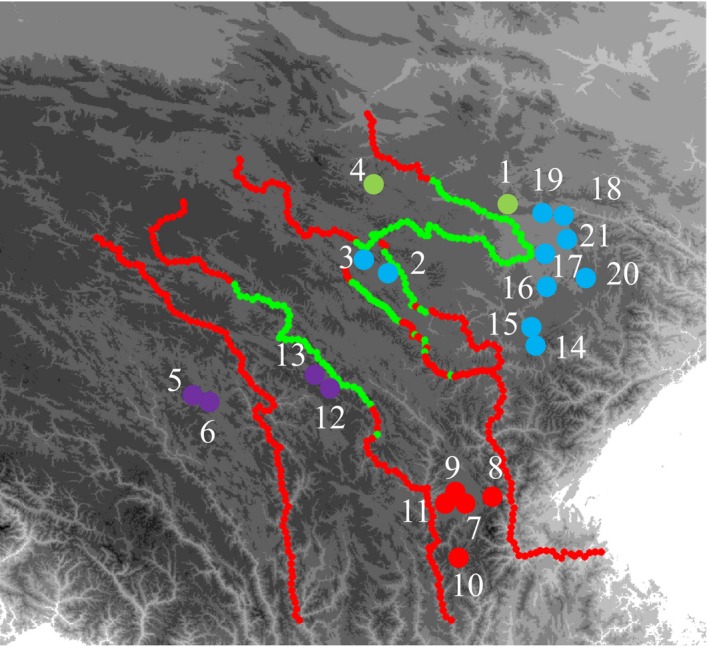
Results of population grouping based on mtDNA data and habitat suitability mapping of rivers. Red, Group S; purple, Group W; green, Group N2; blue, Group N1. Red dash lines indicate unsuitable habitats and green indicates suitable habitats

Population grouping based on mtDNA suggested four groups as FCT reached a plateau at *K* = 4. Localities 7–11 (Matriline S) formed the southern group, Group S. Group S occurs between downstream areas of the Dadu and Yalong rivers. Localities 5, 6, 12, and 13 (Matriline W) comprised Group W, which consists of samples lay west of the Yalong River (Figure 1). The northern region contained two groups. Localities 1 and 4 formed Group N2. The fourth group, Group N1, comprised samples from other localities (mostly N1) (Figure 3). FCT kept increasing with lesser extent when *K* values increase. When setting *K* = 5, FCT reaches 0.876 and localities 8 and 10 were separated from Group S. When setting *K* = 6, localities 12 and 13, which formally belonged to Group W, were suggested as a new group. After setting *K* = 7, locality 21 was separated from Group N1 (Figure S5). Population subdividing was congruent with mtDNA lineage divergence (Figure 1).

**Figure 4:**
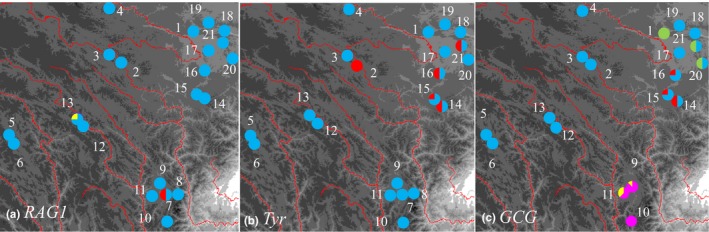
Spatial patterns of nuDNA alleles frequencies. The red lines indicate rivers. Colors indicate different alleles. (a) *RAG‐1*; (b) *Tyr*; (c) *GCG*

Spatial distributions of nuDNA alleles (Figure 4) largely corresponded to major matrilines (Figure 1). There were three alleles in *RAG‐1*. A1 was found in all localities. A2 was only detected in Group S, and A3 was only found in Group W. Only two alleles were recovered in *Tyr*. A1 was widely distributed, and A2 only existed in Group N1. Five alleles were recovered in the intron locus, *GCG*. A2 was the most common allele in groups W, N1, and N2. And two alleles, A1 and A5, were also found in groups N1 and N2. Samples from Group S contained A3 and A4.

**Figure 5:**
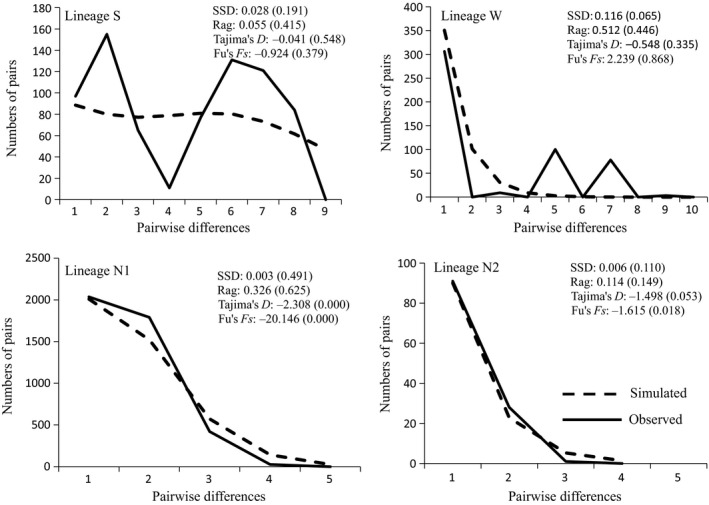
Results of mismatch distribution and neutrality test. Solid lines denote the observed mismatch distributions, and dash lines indicate simulated mismatch distributions. *X*‐axis stands for pairwise differences and *y*‐axis stands for numbers of pairs. The goodness of fit of the observed vs. the theoretical mismatch distributions under a sudden expansion model was tested using the sum of squared deviation and raggedness (Rag) index. Values in brackets indicated *p* values

Mismatch analyses did not include Matriline N3 because a single individual represented it. The analyses did not reject the sudden expansion hypothesis in all populations tested. However, multiple peaks occurred in matrilines S and W (Figure 5). Regarding Tajima's *D* and Fu's *F*s (Figure 5), two matrilines S and W in the sEQTP did not have significantly negative values for either statistics. For northern Matriline N1, both tests were significantly negative. Not significant but negative values of Tajima's *D* and Fu's *F*s were obtained for Matriline N2 (Tajima's *D *=* *−1.49796, *p* = .0527; Fu's *F*s = −1.61464, *p* = .0177).

### 3.5 Species distribution reconstruction

The SDM for N. pleskei differed significantly from the random distribution (*p* < .001). Random null distributions of AUCs ranged from 0.797 to 0.910. In contrast, AUC of SDM based on the real distribution of the species was 0.957.

**Figure 6:**
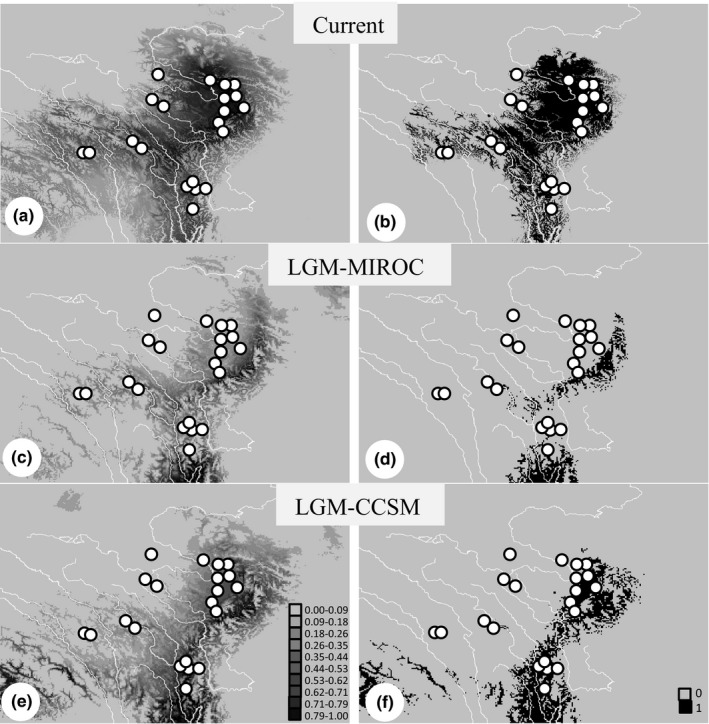
Predicted distribution of *Nanorana pleskei* based on species distribution model. (a) predicted distribution based on current data; (b) predicted current distribution above threshold; (c) distribution during the Last Glacial Maximum (LGM) based on model for interdisciplinary research on climate (MIROC); (d) predicted distribution during LGM based on MIROC above threshold; (e) distribution during the LGM based on community climate system model (CCSM); (f) predicted distribution above the threshold during the LGM based on CCSM. The black lines indicate rivers

The potential distribution of *N. pleskei* was much smaller during the LGM than now based on both CCSM and MIROC (Figure 6). Based on MIROC, the range increased 59% after the LGM. The MIROC suggested a far more dramatic expansion after LGM with an increase of 3.36 times. Both models suggested that northern and southern regions experienced habitat expansion after LGM, albeit at different levels. The CCSM indicated that suitable habitat in the nEQTP increased about 888.8%, but in the sEQTP, only about 117%. The MIROC yielded similar results. The potential distribution in the nEQTP increased 240% and in southern region only 6% (Table 2).

**Table 2:** Potential distribution range changes after LGM. The numbers are grid numbers that are suitable for *Nanorana pleskei* suggested by species distribution model

	Current	LGM‐CCSM	Range increase	LGM‐MIROC	Range increase
All	194,099	44,475	4.364	122,375	1.59
Northern	124,834	12,625	9.888	36,715	3.40
Southern	69,338	31,955	2.170	65,500	1.06

CCSM, community climate system model; LGM, Last Glacial Maximum; MIROC, model for interdisciplinary research on climate.

### 3.6 Barrier effects of rivers

*F*_st_ matrix among different regions is shown in Table 3. All *F*_st_ were significant. Values of *F*_st_ among regions of nEQTP (regions D, E, and F) were lower than sEQTP (regions A, B, and C) (Table 3). Detailed results of dispersal model selection based on BFT are listed in Table 1. Marginal likelihood calculated from raw thermodynamic scores strongly supported model 15 as being the best (*p* > .99). This model assumed that all rivers were barriers that prevented among‐group dispersal for *N. pleskei*, except for the Yellow River. Marginal likelihoods calculated using a Bezier curve did not detect a model significantly outperformed than all other models. Model 9, which assumed the Yalong River and Dadu River were barriers to dispersal, best fits the data with a probability about 0.66. Second best model 2, which assumed the Jinsha River was a barrier for *N. pleskei*, had a probability of about 0.34. All other models had probabilities smaller than 0.01.

**Table 3:** *F*_st_ between six regions separated by rivers. Regions dividing are shown in Figure S1

	E	D	F	A	C
E					
D	0.066				
F	0.560	0.671			
A	0.766	0.913	0.821		
C	0.876	0.891	0.801	0.908	
B	0.672	0.838	0.607	0.906	0.864

**Figure 7:**
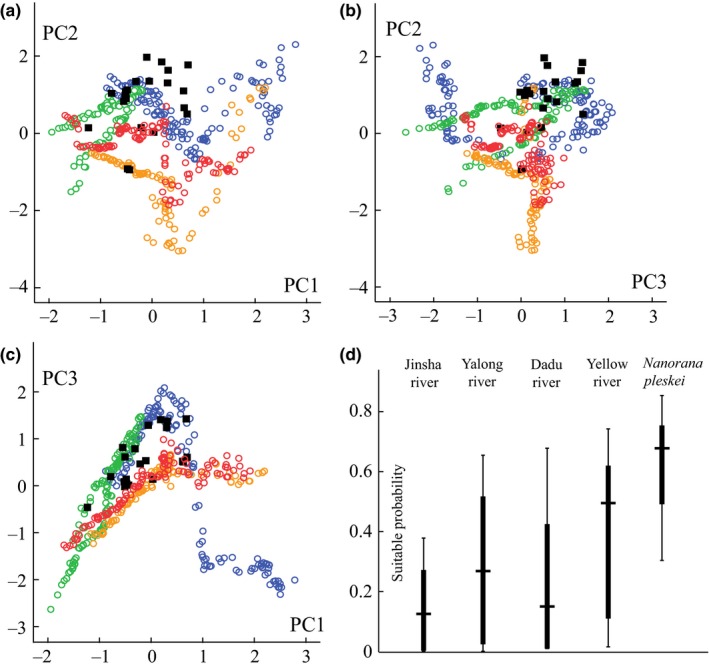
(a–c) Scatter plots of principal components. Black solid squares indicate sampling localities of *Nanorana pleskei*. Empty circles indicate rivers. Purple indicates Dadu River, green indicates Yellow River, orange indicates Jinsha River, and red indicates Yalong River. (d) Overlapping of suitable probability between *N. pleskei* and rivers visualized by box plots

Suitability maps of rivers indicated environments near the rivers were suitable for *N. pleskei* in nEQTP. In contrast, the sEQTP offered unsuitable habitats near rivers, which contributed to the formation of the barrier (Figure 3). Box plots of the value of suitable probability obtained the same patterns (Figure 7). Predicted habitat for *N. pleskei* overlapped with the Yellow River. The suitable probability was lowest along the Jinsha River.

Bioclimatic variables 2, 12, 13, 14, 16, 17, 18, and 19 were deviated from normal distribution. The PCA reduced the bioclimatic variables to three PCs. PC1, PC2, and PC3 explained 58.798%, 23.058%, and 9.176% of the total variance, respectively. The additional components were excluded from subsequent analyses. Scatter plots of these PCs supported that localities of *N. pleskei* differed from extracted points along the rivers. However, they were mixed with parts of the Yellow and Dadu rivers (Figure 7).

## 4 Discussion

### 4.1 Different influences of rivers on *Nanorana pleskei*

Our results reject the null hypotheses that different geographic features in northern EQTP and southern EQTP influenced *N. pleske*i in a similar way. Our analyses demonstrate clearly that different rivers, or different parts of them, differentially influence *N. pleskei*. In the sEQTP, rivers tend to be strong barriers to dispersal for *N. pleskei*, but northern rivers are not effective barriers. Dispersal models indicate ongoing cross‐river dispersals in the nEQTP, but not in the southern areas.

Genetic structures recovered by matrilineal genealogy are different between nEQTP and sEQTP. Three matrilines, including N1, N2, and N3, are found in nEQTP (Figure 1). But the lineages divergences are not corresponding to the rivers. Shared mtDNA haplotypes are detected in localities that separated by the Yellow River and upstream of Dadu river (Figure 1, Table S1). But in sEQTP, matrilineal genealogy divergence pattern consists of rivers. Matriline S is separated from other matrilines by Dadu and Yalong rivers. Matriline W is separated from its sister lineage, Matriline N1, by Yalong River. Two sublineages are recovered within Matriline W, and they are separated by the Jinsha River (Figure 1). *F*_st_ matrix also supports the differences between nEQTP and sEQTP, as *F*_st_ values between regions separated by rivers in nEQTP are lower than those in sEQTP (Table 3). The conservative nuDNA loci provide little information, but the spatial patterns of alleles are not in conflict with mtDNA lineages. In *RAG‐1* gene, two private alleles are detected in matrilines S and W. And in *Tyr*, allele A2 is found in nEQTP and distributes in two sides of the Dadu River. Locus *GCG* contains more information and supports that Matriline S is divergent from other matrilines. Geographically widespread alleles occur in all nuDNA loci. Considering the spatial pattern of mtDNA haplotypes and the physiological characters of amphibian (Beebee, 2005), this pattern probably reflects the persistence of ancestral alleles, not widespread gene flow.

Results from SAMOVA also recover different genetic structure between nEQTP and sEQTP. Two groups, N1 and N2, occur in the nEQTP. They present a pattern not associating with rivers. Group N2 contains two localities (1 and 4), which are separated by the Yellow River (Figure 3). Haplotypes from different matrilines also occur sympatric in localities 4 and 21 (Figure 1). These results indicate recent or ongoing dispersal and gene flow among them. Dispersal modeling supports this scenario, as the models strongly favored by BFT indicate unrestricted mixing among groups in the nEQTP. Two groups, S and W, are in the sEQTP. Dispersal models indicate complete isolation of groups S and W from other groups by rivers. Rivers also promote isolation within groups S and W. Upon setting *K* = 5, Group S is divided into two groups separated by the Xianshui River—a branch of Yalong River (Figure S5). These two subgroups do not share mtDNA haplotypes. Group W consists of two subgroups, which the Jinsha River separates. When setting *K* = 6, they are suggested as two separated groups. Dispersal models indicate each is isolated from all other populations including each other.

Geographic features and environmental characters generate contrasting patterns for the northern EQTP and southern EQTP. The nEQTP contains large areas of marshland along the rivers, and this provides suitable habitat for *N. pleskei*. In contrast, rivers in the sEQTP are much larger than in the north. More importantly, the alpine valleys, instead of marsh, are the most common landscape in the sEQTP (You & Yang, 2013). Furthermore, environment changes drastically from top to the bottom of these valleys. And arid‐hot valley domain covers most part of river valleys in sEQTP. The steep valleys, unsuitable environment, and rapidly flowing rivers offer dispersal barriers for *N. pleskei*. The frogs cannot easily cross mountains and these rivers. Three analyses converge on similar results. After mapping the threshold probability of rivers' suitable habitats, the pattern clearly obtains the same conclusion as the dispersal modeling. On average, habitat suitability is lower for barriers suggested by testing with dispersal models. Scatter plots based on PCs of bioclimate data suggested that riverine environments differed from the sampling localities of *N. pleskei*. Only the Yellow River exhibits some overlap with sampling localities of *N. pleskei*.

The impacts of rivers on amphibians appear to relate to availability of suitable habitat. Deeply incised river valleys can form barriers to dispersal. In contrast, rivers that offer suitable habitats for amphibians promote dispersal, as in the Yellow River in nEQTP. Deeply incised river valleys also can offer refugia for amphibians when suitable habitat persists during harsh times. This situation applies to the closely related species of *N. pleskei* and *N. parkeri*, which occurs commonly in the valleys of Yarlung Zangpo River where several refugia occurred (Zhou et al., 2014). In EQTP, the impacts of rivers are different among species. Rivers in sEQTP are effective barriers for *N. pleskei* and *S. boulengeri* (Li et al., 2009), but not for *R. kukunoris* (Zhao et al., 2009; Zhou et al., 2013). The different impacts may be caused by different characters of these species. Future comprehensive comparison studies can evaluate how rivers in EQTP, especially in the sEQTP, impact other biota.

### 4.2 Demographic history

Our study firmly rejects the null hypothesis that northern and southern populations of *N. pleskei* on the EQTP experience similar demographic histories. Evidences of demographic expansion in northern groups emerge in the neutrality test and mismatch analyses. Neutrality testing supports demographic expansion in the nEQTP group (matrilines N1 and N2 in Figure 1), but not in the sEQTP group (matrilines S and W in Figure 1). Mismatch analyses yield similar results. Although analyses fail to reject significantly the sudden expansion hypothesis in all matrilines, multiple peaks occur in matrilines S and W, which host samples from the sEQTP. In contrast, a single peak exists in the northern matrilines (Figure 5).

Pleistocene habitat changes can explain the differences in demographic history. Large areas of drastic environmental change happen easily in the nEQTP. SDMs of habitat changes following the LGM show clearly that the nEQTP experienced far more severe habitat shifting than the sEQTP, which is supported by both CCSM and MIROC (Figure 6). Geographic features may contribute to the differences in habitat changes. Marshland covers vast areas of the nEQTP and persists homogeneous environment. Large regions can change easily and in doing so drive the rapid demographic expansion–contraction of *N. pleskei*. The complex geographic features that characterize the sEQTP will generate suitable microhabitats, which may serve as micro‐refugia during times of climatic change. For example, although large areas of suitable habitats do not exist west of the Jinsha River, *N. pleskei* occurs there (localities 5 and 6), contains private haplotypes, and remains isolated from other populations. Similar conditions occur at localities 8 and 10. Micro‐refugia helped *N. pleskei* retain population stable.

Our study supports the idea that range and distribution of suitable habitat are the key factors determining demographic histories. The suitable habitats of a species are influenced by both climatic and geographic features. Climate fluctuations have enormous impacts on changes in a species' suitable habitat. But these impacts are influenced by geographic features. Complex geographic features generate higher environmental heterogeneity, which will buffer the habitat shifts and then reduce extent of demographic changes. Such as in our case, *N. pleskei* appears to have retained stable populations in heterogeneous environments but experienced demographic changes in homogeneous ones. Populations in sEQTP retaining stable demographics are not rare. Many studies of species in sEQTP report stable demographics. The rodents *Niviventer excelsior* (Chen et al., 2010), *Neodon irene*, and *Apodemus latronum* (Fan, Liu, Liu, Zhang, & Yue, 2011) do not appear to have experienced demographic expansions in most populations. A comparative study on five avian species supports that two species distributed on the edges of the plateau, the twite (*Carduelis flavirostris*) and the black redstart (*Phoenicurus ochruros*), tended to maintain stable populations (Qu, Lei, Zhang, & Lu, 2010). Notwithstanding, another factor may also contribute to this apparent stability. Climate changes in the QTP were much less severe during the LGM when compared to other glacial periods (Owen, Caffee, Finkel, & Seong, 2008; Schäfer et al., 2002; Zheng, Xu, & Shen, 2002). The LGM imposed very little effect on demographic history of many species, such as the plateau pika (*Ochotona curzoniae*) (Ci et al., 2009), Himalayan hemlock (*Tsuga dumosa*) (Cun & Wang, 2010), and Tibetan antelope (*Pantholops hodgsoni*) (Du et al., 2010). Compared to these species, *N. pleskei* appears to have retained stable populations in heterogeneous environments but experienced demographic changes in homogeneous ones. This result supports the idea that both climatic and geographic features can impact the evolutionary history of a species.

### 4.3 Implications for conservation

*Nanorana pleskei* is considered to be near threatened and decreasing in abundance and number of populations (Wang, Annemarie, Muhammad, & Xie, 2004). A major threat to this species appears to be habitat destruction and degradation caused by overgrazing livestock. Our study discovers significantly different population structure between northern and southern populations of *N. pleskei* in the EQTP. This result has important implications for the conservation of the species. Populations in the sEQTP appear to be disproportionally important for conservation. These isolated populations have experienced stable populations. Genetic evidence can serve to design evolutionarily significant units and management units (Moritz, 1994). Our results indicate that there are multiple management units in the southern region.

The sEQTP belongs to the Hengduan Mountains, a biodiversity hot spot (Myers et al., 2000). Barriers to dispersal and less severe climatic changes greatly influence the genetic structure and demographic history of *N. pleskei*. These factors may also contribute to the high biodiversity in this region, as multiple studies indicate species in this region are isolated by river valleys and retain stable demographics. Our results are not only useful for the conservation of this species, but also for all biota in the EQTP.

## 5 Conclusions

By revealing the genetic structure and population history of *N. pleskei*, our study documents the impacts of different environments of the northern EQTP and southern EQTP on organisms. Rivers in northern EQTP and southern EQTP influence *N. pleskei* in different ways. In the nEQTP, rivers are not barriers but rather provide large patches of continuous habitat that facilitate dispersal, which we detect. However, river valleys and their associated environments in the sEQTP host habitat unsuitable for *N. pleskei*. The demographic histories of *N. pleskei* in northern and southern regions differ and correspond with different geographic features. In the nEQTP, *N. pleskei* experienced demographic expansion, which a homogeneous environment promoted. In contrast, complex geographic features in the sEQTP appear to have generated multiple micro‐refugia.

## Conflict of Interest

None declared.

## Data Accessibility

All sequences were uploaded to GenBank. Bioclimate data are downloaded from the WorldClim database. Details regarding individual samples are available in Table S1.
