# Supplementary material for: Mountains too high and valleys too deep drive population structuring and demographics in a Qinghai–Tibetan Plateau frog Nanorana pleskei (Dicroglossidae)
Source: Ecol Evol. 2016 Dec 18;7(1):240–52. doi: 10.1002/ece3.2646 (PMC5214757; doi:10.1002/ece3.2646)

Table S1: Detailed information for specimens included in this study. KIZ stands for Kunming Institute of Zoology, Chinese Academy of Sciences. Numbers with KIZ stand for sample number and numbers with KIZ-YPX indicates tissue number. Between the brackets are the GenBank accession numbers.

| **Locality number** | **Species name** | **Locality Name** | **Longitude** | **Latitude** | **Elevation (m)** | **Tissue ID** | **Haplotype** | **Genotype of RAG1** | **Genotype of Tyr** | **Genotype of GCG** |
| --- | --- | --- | --- | --- | --- | --- | --- | --- | --- | --- |
| 1 | *Nanorana pleskei* | China: Maqu, Ganan, Gansu Prov. | 102.109°E | 34.077°N | 3749 | KIZ-YPX32889 | H1 (KY172144, KY172327) |  |  |  |
| 1 | *Nanorana pleskei* | China: Maqu, Ganan, Gansu Prov. | 102.109°E | 34.077°N | 3749 | KIZ-YPX32900 | H2 (KY172145, KY172328) | A1A1 (KY172538) |  |  |
| 1 | *Nanorana pleskei* | China: Maqu, Ganan, Gansu Prov. | 102.109°E | 34.077°N | 3749 | KIZ-YPX32958 | H2 (KY172146, KY172329) | A1A1 (KY172539) | A1A1 (KY172593) | A1A1 (KY172510) |
| 1 | *Nanorana pleskei* | China: Maqu, Ganan, Gansu Prov. | 102.109°E | 34.077°N | 3749 | KIZ-YPX32959 | H2 (KY172147, KY172330) | A1A1 (KY172540) |  |  |
| 1 | *Nanorana pleskei* | China: Maqu, Ganan, Gansu Prov. | 102.109°E | 34.077°N | 3749 | KIZ-YPX32960 | H2 (KY172148, KY172331) |  |  |  |
| 1 | *Nanorana pleskei* | China: Maqu, Ganan, Gansu Prov. | 102.109°E | 34.077°N | 3749 | KIZ-YPX32961 | H2 (KY172149, KY172332) |  |  |  |
| 2 | *Nanorana pleskei* | China: Banma, Guoluo, Qinghai Prov. | 100.520°E | 33.190°N | 3950 | KIZ-YPX24321 | H3 (KY172150, KY172333) |  |  |  |
| 2 | *Nanorana pleskei* | China: Banma, Guoluo, Qinghai Prov. | 100.520°E | 33.190°N | 3950 | KIZ-YPX24322 | H4 (KY172151, KY172334) |  |  |  |
| 2 | *Nanorana pleskei* | China: Banma, Guoluo, Qinghai Prov. | 100.520°E | 33.190°N | 3950 | KIZ-YPX24323 | H5 (KY172152, KY172335) |  |  |  |
| 2 | *Nanorana pleskei* | China: Banma, Guoluo, Qinghai Prov. | 100.520°E | 33.190°N | 3950 | KIZ-YPX24324 | H4 (KY172153, KY172336) |  |  |  |
| 2 | *Nanorana pleskei* | China: Banma, Guoluo, Qinghai Prov. | 100.520°E | 33.190°N | 3950 | KIZ-YPX24325 | H3 (KY172154, KY172337) |  |  |  |
| 2 | *Nanorana pleskei* | China: Banma, Guoluo, Qinghai Prov. | 100.520°E | 33.190°N | 3950 | KIZ-YPX25853 | H5 (KY172155, KY172338) | A1A1 (KY172541) | A2A2 (KY172594) | A2A2 (KY172511) |
| 2 | *Nanorana pleskei* | China: Banma, Guoluo, Qinghai Prov. | 100.520°E | 33.190°N | 3950 | KIZ-YPX25854 | H3 (KY172156, KY172339) |  |  |  |
| 2 | *Nanorana pleskei* | China: Banma, Guoluo, Qinghai Prov. | 100.520°E | 33.190°N | 3950 | KIZ-YPX25855 | H5 (KY172157, KY172340) |  |  |  |
| 2 | *Nanorana pleskei* | China: Banma, Guoluo, Qinghai Prov. | 100.520°E | 33.190°N | 3950 | KIZ-YPX25856 | H3 (KY172158, KY172341) |  |  |  |
| 2 | *Nanorana pleskei* | China: Banma, Guoluo, Qinghai Prov. | 100.520°E | 33.190°N | 3950 | KIZ-YPX25857 | H5 (KY172159, KY172342) |  |  |  |
| 2 | *Nanorana pleskei* | China: Banma, Guoluo, Qinghai Prov. | 100.520°E | 33.190°N | 3950 | KIZ-YPX25858 | H4 (KY172160, KY172343) |  |  |  |
| 2 | *Nanorana pleskei* | China: Banma, Guoluo, Qinghai Prov. | 100.520°E | 33.190°N | 3950 | KIZ-YPX25859 | H4 (KY172161, KY172344) | A1A1 (KY172542) |  | A2A2 (KY172512) |
| 2 | *Nanorana pleskei* | China: Banma, Guoluo, Qinghai Prov. | 100.520°E | 33.190°N | 3950 | KIZ-YPX25860 | H3 (KY172162, KY172345) | A1A1 (KY172543) |  |  |
| 2 | *Nanorana pleskei* | China: Banma, Guoluo, Qinghai Prov. | 100.520°E | 33.190°N | 3950 | KIZ-YPX25861 | H3 (KY172163, KY172346) | A1A1 (KY172544) |  |  |
| 3 | *Nanorana pleskei* | China: Dari, Guoluo, Qinghai Prov. | 100.195°E | 33.390°N | 4238 | KIZ020309 | H6 (KY172164, KY172347) |  |  |  |
| 3 | *Nanorana pleskei* | China: Dari, Guoluo, Qinghai Prov. | 100.195°E | 33.390°N | 4238 | KIZ020310 | H3 (KY172165, KY172348) |  |  |  |
| 3 | *Nanorana pleskei* | China: Dari, Guoluo, Qinghai Prov. | 100.195°E | 33.390°N | 4238 | KIZ020311 | H7 (KY172166, KY172349) | A1A1 (KY172545) | A1A1 (KY172595) | |
| 3 | *Nanorana pleskei* | China: Dari, Guoluo, Qinghai Prov. | 100.195°E | 33.390°N | 4238 | KIZ020312 | H3 (KY172167, KY172350) | A1A1 (KY172546) | A1A1 (KY172596) | A2A2 (KY172513) |
| 3 | *Nanorana pleskei* | China: Dari, Guoluo, Qinghai Prov. | 100.195°E | 33.390°N | 4238 | KIZ020314 | H3 (KY172168, KY172351) |  |  |  |
| 3 | *Nanorana pleskei* | China: Dari, Guoluo, Qinghai Prov. | 100.195°E | 33.390°N | 4238 | KIZ-YPX24446 | H8 (KY172169, KY172352) |  |  |  |
| 3 | *Nanorana pleskei* | China: Dari, Guoluo, Qinghai Prov. | 100.195°E | 33.390°N | 4238 | KIZ-YPX24448 | H3 (KY172170, KY172353) |  |  |  |
| 3 | *Nanorana pleskei* | China: Dari, Guoluo, Qinghai Prov. | 100.195°E | 33.390°N | 4238 | KIZ-YPX24451 | H7 (KY172171, KY172354) |  |  |  |
| 3 | *Nanorana pleskei* | China: Dari, Guoluo, Qinghai Prov. | 100.195°E | 33.390°N | 4238 | KIZ-YPX24452 | H9 (KY172172, KY172355) |  |  |  |
| 4 | *Nanorana pleskei* | China: Maduo, Guoluo, Qinghai Prov. | 100.256°E | 34.369°N | 3889 | KIZ020182 | H10 (KY172173, KY172356) | A1A1 (KY172547) | A1A1 (KY172597) | |
| 4 | *Nanorana pleskei* | China: Maduo, Guoluo, Qinghai Prov. | 100.256°E | 34.369°N | 3889 | KIZ020183 | H2 (KY172174, KY172357) |  |  |  |
| 4 | *Nanorana pleskei* | China: Maduo, Guoluo, Qinghai Prov. | 100.256°E | 34.369°N | 3889 | KIZ020184 | H11 (KY172175, KY172358) | A1A1 (KY172548) | A1A1 (KY172598) | |
| 4 | *Nanorana pleskei* | China: Maduo, Guoluo, Qinghai Prov. | 100.256°E | 34.369°N | 3889 | KIZ020185 | H2 (KY172176, KY172359) |  |  |  |
| 4 | *Nanorana pleskei* | China: Maduo, Guoluo, Qinghai Prov. | 100.256°E | 34.369°N | 3889 | KIZ-YPX24285 | H3 (KY172177, KY172360) | A1A1 (KY172549) |  |  |
| 4 | *Nanorana pleskei* | China: Maduo, Guoluo, Qinghai Prov. | 100.256°E | 34.369°N | 3889 | KIZ-YPX24287 | H2 (KY172178, KY172361) |  |  |  |
| 4 | *Nanorana pleskei* | China: Maduo, Guoluo, Qinghai Prov. | 100.256°E | 34.369°N | 3889 | KIZ-YPX24288 | H2 (KY172179, KY172362) |  |  |  |
| 4 | *Nanorana pleskei* | China: Maduo, Guoluo, Qinghai Prov. | 100.256°E | 34.369°N | 3889 | KIZ-YPX24289 | H2 (KY172180, KY172363) | A1A1 (KY172550) | A1A1 (KY172599) | A2A2 (KY172514) |
| 4 | *Nanorana pleskei* | China: Maduo, Guoluo, Qinghai Prov. | 100.256°E | 34.369°N | 3889 | KIZ-YPX24291 | H12 (KY172181, KY172364) | A1A1 (KY172551) |  |  |
| 4 | *Nanorana pleskei* | China: Maduo, Guoluo, Qinghai Prov. | 100.256°E | 34.369°N | 3889 | KIZKIZ-YPX24378 | H2 (KY172182, KY172365) |  |  |  |
| 5 | *Nanorana pleskei* | China: Jiangda, Changdu, Tibetan Autonomous Region | 97.853°E | 31.395°N | 4084 | KIZ014906 | H13 (KY172183, KY172366) | A1A1 (KY172552) | A1A1 (KY172600) | A2A2 (KY172515) |
| 5 | *Nanorana pleskei* | China: Jiangda, Changdu, Tibetan Autonomous Region | 97.853°E | 31.395°N | 4084 | KIZ014907 | H13 (KY172184, KY172367) |  |  |  |
| 5 | *Nanorana pleskei* | China: Jiangda, Changdu, Tibetan Autonomous Region | 97.853°E | 31.395°N | 4084 | KIZ014908 | H14 (KY172185, KY172368) |  |  |  |
| 5 | *Nanorana pleskei* | China: Jiangda, Changdu, Tibetan Autonomous Region | 97.853°E | 31.395°N | 4084 | KIZ014909 | H13 (KY172186, KY172369) |  |  |  |
| 5 | *Nanorana pleskei* | China: Jiangda, Changdu, Tibetan Autonomous Region | 97.853°E | 31.395°N | 4084 | KIZ-YPX28510 | H13 (KY172187, KY172370) |  |  |  |
| 5 | *Nanorana pleskei* | China: Jiangda, Changdu, Tibetan Autonomous Region | 97.853°E | 31.395°N | 4084 | KIZ-YPX28511 | H13 (KY172188, KY172371) |  |  |  |
| 5 | *Nanorana pleskei* | China: Jiangda, Changdu, Tibetan Autonomous Region | 97.853°E | 31.395°N | 4084 | KIZ-YPX28521 | H13 (KY172189, KY172372) |  |  |  |
| 5 | *Nanorana pleskei* | China: Jiangda, Changdu, Tibetan Autonomous Region | 97.853°E | 31.395°N | 4084 | KIZ-YPX28522 | H13 (KY172190, KY172373) |  |  |  |
| 5 | *Nanorana pleskei* | China: Jiangda, Changdu, Tibetan Autonomous Region | 97.853°E | 31.395°N | 4084 | KIZ-YPX28523 | H13 (KY172191, KY172374) |  |  |  |
| 5 | *Nanorana pleskei* | China: Jiangda, Changdu, Tibetan Autonomous Region | 97.853°E | 31.395°N | 4084 | KIZ-YPX28524 | H13 (KY172192, KY172375) |  |  |  |
| 5 | *Nanorana pleskei* | China: Jiangda, Changdu, Tibetan Autonomous Region | 97.853°E | 31.395°N | 4084 | KIZ-YPX28525 | H13 (KY172193, KY172376) |  |  |  |
| 5 | *Nanorana pleskei* | China: Jiangda, Changdu, Tibetan Autonomous Region | 97.853°E | 31.395°N | 4084 | KIZ-YPX28526 | H13 (KY172194, KY172377) |  |  |  |
| 6 | *Nanorana pleskei* | China: Jiangda, Changdu, Tibetan Autonomous Region | 97.899°E | 31.389°N | 3669 | KIZ-YPX30869 | H13 (KY172195, KY172378) |  |  |  |
| 6 | *Nanorana pleskei* | China: Jiangda, Changdu, Tibetan Autonomous Region | 97.899°E | 31.389°N | 3669 | KIZ-YPX30870 | H13 (KY172196, KY172379) |  |  |  |
| 6 | *Nanorana pleskei* | China: Jiangda, Changdu, Tibetan Autonomous Region | 97.899°E | 31.389°N | 3669 | KIZ-YPX30871 | H13 (KY172197, KY172380) |  |  |  |
| 6 | *Nanorana pleskei* | China: Jiangda, Changdu, Tibetan Autonomous Region | 97.899°E | 31.389°N | 3669 | KIZ-YPX30872 | H13 (KY172198, KY172381) |  |  |  |
| 6 | *Nanorana pleskei* | China: Jiangda, Changdu, Tibetan Autonomous Region | 97.899°E | 31.389°N | 3669 | KIZ-YPX30873 | H13 (KY172199, KY172382) |  |  |  |
| 6 | *Nanorana pleskei* | China: Jiangda, Changdu, Tibetan Autonomous Region | 97.899°E | 31.389°N | 3669 | KIZ-YPX30874 | H13 (KY172200, KY172383) |  |  |  |
| 6 | *Nanorana pleskei* | China: Jiangda, Changdu, Tibetan Autonomous Region | 97.899°E | 31.389°N | 3669 | KIZ-YPX30875 | H13 (KY172201, KY172384) |  |  |  |
| 6 | *Nanorana pleskei* | China: Jiangda, Changdu, Tibetan Autonomous Region | 97.899°E | 31.389°N | 3669 | KIZ-YPX30876 | H13 (KY172202, KY172385) |  |  |  |
| 6 | *Nanorana pleskei* | China: Jiangda, Changdu, Tibetan Autonomous Region | 97.899°E | 31.389°N | 3669 | KIZ-YPX30877 | H13 (KY172203, KY172386) |  |  |  |
| 6 | *Nanorana pleskei* | China: Jiangda, Changdu, Tibetan Autonomous Region | 97.899°E | 31.389°N | 3669 | KIZ-YPX30878 | H13 (KY172204, KY172387) |  |  |  |
| 6 | *Nanorana pleskei* | China: Jiangda, Changdu, Tibetan AutonomousRegion | 97.899°E | 31.389°N | 3669 | KIZ-YPX30879 | H13 (KY172205, KY172388) |  |  |  |
| 6 | *Nanorana pleskei* | China: Jiangda, Changdu, Tibetan Autonomous Region | 97.899°E | 31.389°N | 3669 | KIZ-YPX30880 | H13 (KY172206, KY172389) | A1A1 (KY172553) | A1A1 (KY172601) | A2A2 (KY172516) |
| 6 | *Nanorana pleskei* | China: Jiangda, Changdu, Tibetan Autonomous Region | 97.899°E | 31.389°N | 3669 | KIZ-YPX30881 | H13 (KY172207, KY172390) |  |  |  |
| 6 | *Nanorana pleskei* | China: Jiangda, Changdu, Tibetan Autonomous Region | 97.899°E | 31.389°N | 3669 | KIZ-YPX30882 | H13 (KY172208, KY172391) |  |  |  |
| 7 | *Nanorana pleskei* | China: Xinduqiao, Kangding, Ganzi, Sichuan Prov. | 101.541°E | 30.036°N | 3611 | KIZ-YPX32439 | H15 (KY172209, KY172392) | A1A2 (KY172554) | A1A1 (KY172602) | |
| 8 | *Nanorana pleskei* | China: Zheduoshan, Kangding, Ganzi, Sichuan Prov. | 101.852°E | 30.029°N | 3693 | KIZ045001 | H16 (KY172210, KY172393) | A1A1 (KY172555) | A1A1 (KY172603) | |
| 8 | *Nanorana pleskei* | China: Zheduoshan, Kangding, Ganzi, Sichuan Prov. | 101.852°E | 30.029°N | 3695 | KIZ045002 | H17 (KY172211, KY172394) | A1A1 (KY172556) | A1A1 (KY172604) | |
| 9 | *Nanorana pleskei* | China: Kangding, Ganzi, Sichuan Prov. | 101.479°E | 30.099°N | 3523 | KIZ045003 | H18 (KY172212, KY172395) |  |  |  |
| 9 | *Nanorana pleskei* | China: Kangding, Ganzi, Sichuan Prov. | 101.479°E | 30.099°N | 3523 | KIZ-YPX32397 | H18 (KY172213, KY172396) |  |  |  |
| 9 | *Nanorana pleskei* | China: Kangding, Ganzi, Sichuan Prov. | 101.479°E | 30.099°N | 3523 | KIZ045006 | H19 (KY172214, KY172397) |  |  |  |
| 9 | *Nanorana pleskei* | China: Kangding, Ganzi, Sichuan Prov. | 101.479°E | 30.099°N | 3523 | KIZ045008 | H19 (KY172215, KY172398) |  |  |  |
| 9 | *Nanorana pleskei* | China: Kangding, Ganzi, Sichuan Prov. | 101.479°E | 30.099°N | 3523 | KIZ045009 | H19 (KY172216, KY172399) |  |  |  |
| 9 | *Nanorana pleskei* | China: Kangding, Ganzi, Sichuan Prov. | 101.479°E | 30.099°N | 3523 | KIZ-YPX32402 | H19 (KY172217, KY172400) |  |  |  |
| 9 | *Nanorana pleskei* | China: Kangding, Ganzi, Sichuan Prov. | 101.479°E | 30.099°N | 3523 | KIZ-YPX32403 | H18 (KY172218, KY172401) | A1A1 (KY172557) | A1A1 (KY172605) | A3A3 (KY172517) |
| 9 | *Nanorana pleskei* | China: Kangding, Ganzi, Sichuan Prov. | 101.479°E | 30.099°N | 3523 | KIZ-YPX32407 | H20 (KY172219, KY172402) | A1A1 (KY172558) | A1A1 (KY172606) | A3A4 (KY172518) |
| 9 | *Nanorana pleskei* | China: Kangding, Ganzi, Sichuan Prov. | 101.479°E | 30.099°N | 3523 | KIZ-YPX32408 | H19 (KY172220, KY172403) | A1A1 (KY172559) | A1A1 (KY172607) | A3A3 (KY172519) |
| 9 | *Nanorana pleskei* | China: Kangding, Ganzi, Sichuan Prov. | 101.479°E | 30.099°N | 3523 | KIZ-YPX32411 | H21 (KY172221, KY172404) | A1A1 (KY172560) | A1A1 (KY172608) | A3A3 (KY172520) |
| 9 | *Nanorana pleskei* | China: Kangding, Ganzi, Sichuan Prov. | 101.479°E | 30.099°N | 3523 | KIZ-YPX32412 | H19 (KY172222, KY172405) | A1A1 (KY172561) |  |  |
| 9 | *Nanorana pleskei* | China: Kangding, Ganzi, Sichuan Prov. | 101.479°E | 30.099°N | 3523 | KIZ-YPX32413 | H19 (KY172223, KY172406) | A1A1 (KY172562) |  |  |
| 10 | *Nanorana pleskei* | China:Jiulong, Ganzi, Sichuan Prov. | 101.454°E | 29.227°N | 3779 | KIZ045047 | H16 (KY172224, KY172407) | A1A1 (KY172563) | A1A1 (KY172609) | A3A3 (KY172521) |
| 10 | *Nanorana pleskei* | China:Jiulong, Ganzi, Sichuan Prov. | 101.454°E | 29.227°N | 3779 | KIZ045048 | H16 (KY172225, KY172408) | A1A1 (KY172564) |  |  |
| 10 | *Nanorana pleskei* | China:Jiulong, Ganzi, Sichuan Prov. | 101.454°E | 29.227°N | 3779 | KIZ045049 | H22 (KY172226, KY172409) |  |  |  |
| 11 | *Nanorana pleskei* | China: Kangding, Ganzi, Sichuan Prov. | 101.373°E | 30.051°N | 4284 | KIZ045053 | H21 (KY172227, KY172410) | A1A1 (KY172565) | A1A1 (KY172610) | A4A4 (KY172522) |
| 11 | *Nanorana pleskei* | China: Kangding, Ganzi, Sichuan Prov. | 101.373°E | 30.051°N | 4284 | KIZ045054 | H23 (KY172228, KY172411) | A1A1 (KY172566) | A1A1 (KY172611) | A3A4 (KY172523) |
| 11 | *Nanorana pleskei* | China: Kangding, Ganzi, Sichuan Prov. | 101.373°E | 30.051°N | 4284 | KIZ045103 | H21 (KY172229, KY172412) |  |  |  |
| 11 | *Nanorana pleskei* | China: Kangding, Ganzi, Sichuan Prov. | 101.373°E | 30.051°N | 4284 | KIZ045104 | H21 (KY172230, KY172413) |  |  |  |
| 11 | *Nanorana pleskei* | China: Kangding, Ganzi, Sichuan Prov. | 101.373°E | 30.051°N | 4284 | KIZ-YPX32564 | H24 (KY172231, KY172414) |  |  |  |
| 11 | *Nanorana pleskei* | China: Kangding, Ganzi, Sichuan Prov. | 101.373°E | 30.051°N | 4284 | KIZ-YPX32565 | H23 (KY172232, KY172415) |  |  |  |
| 11 | *Nanorana pleskei* | China: Kangding, Ganzi, Sichuan Prov. | 101.373°E | 30.051°N | 4284 | KIZ-YPX32566 | H21 (KY172233, KY172416) |  |  |  |
| 11 | *Nanorana pleskei* | China: Kangding, Ganzi, Sichuan Prov. | 101.373°E | 30.051°N | 4284 | KIZ-YPX32567 | H25 (KY172234, KY172417) | A1A1 (KY172567) | A1A1 (KY172612) | A3A3 (KY172524) |
| 11 | *Nanorana pleskei* | China: Kangding, Ganzi, Sichuan Prov. | 101.373°E | 30.051°N | 4284 | KIZ-YPX32568 | H23 (KY172235, KY172418) |  |  |  |
| 11 | *Nanorana pleskei* | China: Kangding, Ganzi, Sichuan Prov. | 101.373°E | 30.051°N | 4284 | KIZ-YPX32569 | H21 (KY172236, KY172419) |  |  |  |
| 11 | *Nanorana pleskei* | China: Kangding, Ganzi, Sichuan Prov. | 101.373°E | 30.051°N | 4284 | KIZ-YPX32571 | H21 (KY172237, KY172420) |  |  |  |
| 11 | *Nanorana pleskei* | China: Kangding, Ganzi, Sichuan Prov. | 101.373°E | 30.051°N | 4284 | KIZ-YPX32572 | H25 (KY172238, KY172421) | A1A1 (KY172568) |  |  |
| 11 | *Nanorana pleskei* | China: Kangding, Ganzi, Sichuan Prov. | 101.373°E | 30.051°N | 4284 | KIZ-YPX32573 | H24 (KY172239, KY172422) |  |  |  |
| 11 | *Nanorana pleskei* | China: Kangding, Ganzi, Sichuan Prov. | 101.373°E | 30.051°N | 4284 | KIZ-YPX32574 | H23 (KY172240, KY172423) |  |  |  |
| 11 | *Nanorana pleskei* | China: Kangding, Ganzi, Sichuan Prov. | 101.373°E | 30.051°N | 4284 | KIZ-YPX32575 | H21 (KY172241, KY172424) | A1A1 (KY172569) | A1A1 (KY172613) | A3A3 (KY172525) |
| 11 | *Nanorana pleskei* | China: Kangding, Ganzi, Sichuan Prov. | 101.373°E | 30.051°N | 4284 | KIZ-YPX32576 | H21 (KY172242, KY172425) | A1A1 (KY172570) |  |  |
| 11 | *Nanorana pleskei* | China: Kangding, Ganzi, Sichuan Prov. | 101.373°E | 30.051°N | 4284 | KIZ-YPX32577 | H23 (KY172243, KY172426) |  |  |  |
| 11 | *Nanorana pleskei* | China: Kangding, Ganzi, Sichuan Prov. | 101.373°E | 30.051°N | 4284 | KIZ-YPX32578 | H23 (KY172244, KY172427) |  |  |  |
| 11 | *Nanorana pleskei* | China: Kangding, Ganzi, Sichuan Prov. | 101.373°E | 30.051°N | 4284 | KIZ-YPX32579 | H24 (KY172245, KY172428) |  |  |  |
| 11 | *Nanorana pleskei* | China: Kangding, Ganzi, Sichuan Prov. | 101.373°E | 30.051°N | 4284 | KIZ-YPX32580 | H21 (KY172246, KY172429) | A1A1 (KY172571) |  |  |
| 11 | *Nanorana pleskei* | China: Kangding, Ganzi, Sichuan Prov. | 101.373°E | 30.051°N | 4284 | KIZ-YPX32581 | H23 (KY172247, KY172430) |  |  |  |
| 12 | *Nanorana pleskei* | China: Ganzi, Ganzi, Sichuan Prov. | 99.679°E | 31.679°N | 3568 | KIZ045153 | H26 (KY172248, KY172431) |  |  |  |
| 12 | *Nanorana pleskei* | China: Ganzi, Ganzi, Sichuan Prov. | 99.679°E | 31.679°N | 3568 | KIZ045154 | H26 (KY172249, KY172432) |  |  |  |
| 12 | *Nanorana pleskei* | China: Ganzi, Ganzi, Sichuan Prov. | 99.679°E | 31.679°N | 3568 | KIZ045155 | H26 (KY172250, KY172433) | A1A1 (KY172572) | A1A1 (KY172614) | A2A2 (KY172526) |
| 13 | *Nanorana pleskei* | China: Ganzi, Ganzi, Sichuan Prov. | 99.534°E | 31.760°N | 4022 | KIZ045150 | H27 (KY172251, KY172434) | A1A3 (KY172573) | A1A1 (KY172615) | A2A2 (KY172527) |
| 13 | *Nanorana pleskei* | China: Ganzi, Ganzi, Sichuan Prov. | 99.534°E | 31.760°N | 4022 | KIZ045151 | H27 (KY172252, KY172435) | A1A1 (KY172574) |  |  |
| 13 | *Nanorana pleskei* | China: Ganzi, Ganzi, Sichuan Prov. | 99.534°E | 31.760°N | 4022 | KIZ045152 | H27 (KY172253, KY172436) |  |  |  |
| 14 | *Nanorana pleskei* | China: Hongyuan, Aba, Sichuan Prov. | 102.491°E | 32.287°N | 3620 | KIZ045185 | H3 (KY172254, KY172437) | A1A1 (KY172575) | A1A2 (KY172616) | A2A5 (KY172528) |
| 15 | *Nanorana pleskei* | China: Hongyuan, Aba, Sichuan Prov. | 102.443°E | 32.348°N | 3815 | KIZ045204 | H3 (KY172255, KY172438) | A1A1 (KY172576) |  |  |
| 15 | *Nanorana pleskei* | China: Hongyuan, Aba, Sichuan Prov. | 102.443°E | 32.348°N | 3815 | KIZ045205 | H8 (KY172256, KY172439) | A1A1 (KY172577) |  |  |
| 15 | *Nanorana pleskei* | China: Hongyuan, Aba, Sichuan Prov. | 102.443°E | 32.348°N | 3815 | KIZ045206 | H3 (KY172257, KY172440) | A1A1 (KY172578) | A2A2 (KY172617) | A2A2 (KY172529) |
| 15 | *Nanorana pleskei* | China: Hongyuan, Aba, Sichuan Prov. | 102.443°E | 32.348°N | 3815 | KIZ045208 | H3 (KY172258, KY172441) |  |  |  |
| 15 | *Nanorana pleskei* | China: Hongyuan, Aba, Sichuan Prov. | 102.443°E | 32.348°N | 3815 | KIZ-YPX32736 | H3 (KY172259, KY172442) |  |  |  |
| 15 | *Nanorana pleskei* | China: Hongyuan, Aba, Sichuan Prov. | 102.443°E | 32.348°N | 3815 | KIZ-YPX32737 | H3 (KY172260, KY172443) |  |  |  |
| 15 | *Nanorana pleskei* | China: Hongyuan, Aba, Sichuan Prov. | 102.443°E | 32.348°N | 3815 | KIZ-YPX32738 | H3 (KY172261, KY172444) |  |  |  |
| 15 | *Nanorana pleskei* | China: Hongyuan, Aba, Sichuan Prov. | 102.443°E | 32.348°N | 3815 | KIZ-YPX32739 | H3 (KY172262, KY172445) |  |  |  |
| 15 | *Nanorana pleskei* | China: Hongyuan, Aba, Sichuan Prov. | 102.443°E | 32.348°N | 3815 | KIZ-YPX32740 | H3 (KY172263, KY172446) |  |  |  |
| 15 | *Nanorana pleskei* | China: Hongyuan, Aba, Sichuan Prov. | 102.443°E | 32.348°N | 3815 | KIZ-YPX32741 | H3 (KY172264, KY172447) |  |  |  |
| 15 | *Nanorana pleskei* | China: Hongyuan, Aba, Sichuan Prov. | 102.443°E | 32.348°N | 3815 | KIZ-YPX32743 | H3 (KY172265, KY172448) |  |  |  |
| 15 | *Nanorana pleskei* | China: Hongyuan, Aba, Sichuan Prov. | 102.443°E | 32.348°N | 3815 | KIZ-YPX32744 | H28 (KY172266, KY172449) |  |  |  |
| 15 | *Nanorana pleskei* | China: Hongyuan, Aba, Sichuan Prov. | 102.443°E | 32.348°N | 3815 | KIZ-YPX32748 | H29 (KY172267, KY172450) | A1A1 (KY172579) | A1A2 (KY172618) | A2A5 (KY172530) |
| 15 | *Nanorana pleskei* | China: Hongyuan, Aba, Sichuan Prov. | 102.443°E | 32.348°N | 3815 | KIZ-YPX32749 | H3 (KY172268, KY172451) |  |  |  |
| 15 | *Nanorana pleskei* | China: Hongyuan, Aba, Sichuan Prov. | 102.443°E | 32.348°N | 3815 | KIZ-YPX32750 | H3 (KY172269, KY172452) |  |  |  |
| 16 | *Nanorana pleskei* | China: Hongyuan, Aba, Sichuan Prov. | 102.615°E | 32.994°N | 3469 | KIZ045231 | H29 (KY172270, KY172453) |  |  |  |
| 16 | *Nanorana pleskei* | China: Hongyuan, Aba, Sichuan Prov. | 102.615°E | 32.994°N | 3469 | KIZ045232 | H3 (KY172271, KY172454) | A1A1 (KY172580) | A1A2 (KY172619) | A2A2 (KY172531) |
| 16 | *Nanorana pleskei* | China: Hongyuan, Aba, Sichuan Prov. | 102.615°E | 32.994°N | 3469 | KIZ045233 | H3 (KY172272, KY172455) |  |  |  |
| 16 | *Nanorana pleskei* | China: Hongyuan, Aba, Sichuan Prov. | 102.615°E | 32.994°N | 3469 | KIZ045234 | H3 (KY172273, KY172456) |  |  |  |
| 16 | *Nanorana pleskei* | China: Hongyuan, Aba, Sichuan Prov. | 102.615°E | 32.994°N | 3469 | KIZ-YPX32763 | H3 (KY172274, KY172457) |  |  |  |
| 16 | *Nanorana pleskei* | China: Hongyuan, Aba, Sichuan Prov. | 102.615°E | 32.994°N | 3469 | KIZ-YPX32766 | H30 (KY172275, KY172458) |  |  |  |
| 16 | *Nanorana pleskei* | China: Hongyuan, Aba, Sichuan Prov. | 102.615°E | 32.994°N | 3469 | KIZ-YPX32767 | H29 (KY172276, KY172459) | A1A1 (KY172581) | A1A2 (KY172620) | A2A5 (KY172532) |
| 16 | *Nanorana pleskei* | China: Hongyuan, Aba, Sichuan Prov. | 102.615°E | 32.994°N | 3469 | KIZ-YPX32769 | H3 (KY172277, KY172460) |  |  |  |
| 16 | *Nanorana pleskei* | China: Hongyuan, Aba, Sichuan Prov. | 102.615°E | 32.994°N | 3469 | KIZ-YPX32770 | H3 (KY172278, KY172461) |  |  |  |
| 16 | *Nanorana pleskei* | China: Hongyuan, Aba, Sichuan Prov. | 102.615°E | 32.994°N | 3469 | KIZ-YPX32771 | H3 (KY172279, KY172462) |  |  |  |
| 16 | *Nanorana pleskei* | China: Hongyuan, Aba, Sichuan Prov. | 102.615°E | 32.994°N | 3469 | KIZ-YPX32778 | H31 (KY172280, KY172463) |  |  |  |
| 16 | *Nanorana pleskei* | China: Hongyuan, Aba, Sichuan Prov. | 102.615°E | 32.994°N | 3469 | KIZ-YPX32780 | H30 (KY172281, KY172464) |  |  |  |
| 16 | *Nanorana pleskei* | China: Hongyuan, Aba, Sichuan Prov. | 102.615°E | 32.994°N | 3469 | KIZ-YPX32781 | H3 (KY172282, KY172465) |  |  |  |
| 17 | *Nanorana pleskei* | China: Hongyuan, Aba, Sichuan Prov. | 102.630°E | 33.444°N | 3498 | KIZ045261 | H3 (KY172283, KY172466) | A1A1 (KY172582) |  | A2A2 (KY172533) |
| 17 | *Nanorana pleskei* | China: Hongyuan, Aba, Sichuan Prov. | 102.630°E | 33.444°N | 3498 | KIZ045262 | H3 (KY172284, KY172467) |  |  |  |
| 17 | *Nanorana pleskei* | China: Hongyuan, Aba, Sichuan Prov. | 102.630°E | 33.444°N | 3498 | KIZ045263 | H3 (KY172285, KY172468) |  |  |  |
| 17 | *Nanorana pleskei* | China: Hongyuan, Aba, Sichuan Prov. | 102.630°E | 33.444°N | 3498 | KIZ045264 | H32 (KY172286, KY172469) |  |  |  |
| 17 | *Nanorana pleskei* | China: Hongyuan, Aba, Sichuan Prov. | 102.630°E | 33.444°N | 3498 | KIZ-YPX32792 | H3 (KY172287, KY172470) |  |  |  |
| 17 | *Nanorana pleskei* | China: Hongyuan, Aba, Sichuan Prov. | 102.630°E | 33.444°N | 3498 | KIZ-YPX32793 | H3 (KY172288, KY172471) |  |  |  |
| 17 | *Nanorana pleskei* | China: Hongyuan, Aba, Sichuan Prov. | 102.630°E | 33.444°N | 3498 | KIZ-YPX32794 | H3 (KY172289, KY172472) |  |  |  |
| 17 | *Nanorana pleskei* | China: Hongyuan, Aba, Sichuan Prov. | 102.630°E | 33.444°N | 3498 | KIZ-YPX32795 | H3 (KY172290, KY172473) |  |  |  |
| 17 | *Nanorana pleskei* | China: Hongyuan, Aba, Sichuan Prov. | 102.630°E | 33.444°N | 3498 | KIZ-YPX32796 | H3 (KY172291, KY172474) |  |  |  |
| 17 | *Nanorana pleskei* | China: Hongyuan, Aba, Sichuan Prov. | 102.630°E | 33.444°N | 3498 | KIZ-YPX32797 | H3 (KY172292, KY172475) |  |  |  |
| 17 | *Nanorana pleskei* | China: Hongyuan, Aba, Sichuan Prov. | 102.630°E | 33.444°N | 3498 | KIZ-YPX32798 | H32 (KY172293, KY172476) |  |  |  |
| 17 | *Nanorana pleskei* | China: Hongyuan, Aba, Sichuan Prov. | 102.630°E | 33.444°N | 3498 | KIZ-YPX32799 | H33 (KY172294, KY172477) |  |  |  |
| 17 | *Nanorana pleskei* | China: Hongyuan, Aba, Sichuan Prov. | 102.630°E | 33.444°N | 3498 | KIZ-YPX32800 | H3 (KY172295, KY172478) |  |  |  |
| 17 | *Nanorana pleskei* | China: Hongyuan, Aba, Sichuan Prov. | 102.630°E | 33.444°N | 3498 | KIZ-YPX32801 | H3 (KY172296, KY172479) |  |  |  |
| 17 | *Nanorana pleskei* | China: Hongyuan, Aba, Sichuan Prov. | 102.630°E | 33.444°N | 3498 | KIZ-YPX32804 | H3 (KY172297, KY172480) |  |  |  |
| 17 | *Nanorana pleskei* | China: Hongyuan, Aba, Sichuan Prov. | 102.630°E | 33.444°N | 3498 | KIZ-YPX32805 | H3 (KY172298, KY172481) |  |  |  |
| 18 | *Nanorana pleskei* | China: Ruoergai, Aba, Sichuan Prov. | 102.878°E | 33.928°N | 3467 | KIZ045279 | H3 (KY172299, KY172482) | A1A1 (KY172583) | A1A1 (KY172621) | A2A2 (KY172534) |
| 18 | *Nanorana pleskei* | China: Ruoergai, Aba, Sichuan Prov. | 102.878°E | 33.928°N | 3467 | KIZ045283 | H3 (KY172300, KY172483) |  |  |  |
| 18 | *Nanorana pleskei* | China: Ruoergai, Aba, Sichuan Prov. | 102.878°E | 33.928°N | 3467 | KIZ045284 | H3 (KY172301, KY172484) | A1A1 (KY172584) |  |  |
| 19 | *Nanorana pleskei* | China: Ruoergai, Aba, Sichuan Prov. | 102.695°E | 33.990°N | 3450 | KIZ045295 | H3 (KY172302, KY172485) | A1A1 (KY172585) |  |  |
| 19 | *Nanorana pleskei* | China: Ruoergai, Aba, Sichuan Prov. | 102.695°E | 33.990°N | 3450 | KIZ045296 | H3 (KY172303, KY172486) | A1A1 (KY172586) | A1A1 (KY172622) | A2A2 (KY172535) |
| 19 | *Nanorana pleskei* | China: Ruoergai, Aba, Sichuan Prov. | 102.695°E | 33.990°N | 3450 | KIZ045297 | H3 (KY172304, KY172487) |  |  |  |
| 19 | *Nanorana pleskei* | China: Ruoergai, Aba, Sichuan Prov. | 102.695°E | 33.990°N | 3450 | KIZ045298 | H34 (KY172305, KY172488) |  |  |  |
| 19 | *Nanorana pleskei* | China: Ruoergai, Aba, Sichuan Prov. | 102.695°E | 33.990°N | 3450 | KIZ-YPX32835 | H35 (KY172306, KY172489) |  |  |  |
| 19 | *Nanorana pleskei* | China: Ruoergai, Aba, Sichuan Prov. | 102.695°E | 33.990°N | 3450 | KIZ-YPX32836 | H36 (KY172307, KY172490) |  |  |  |
| 19 | *Nanorana pleskei* | China: Ruoergai, Aba, Sichuan Prov. | 102.695°E | 33.990°N | 3450 | KIZ-YPX32839 | H36 (KY172308, KY172491) |  |  |  |
| 20 | *Nanorana pleskei* | China: Hongyuan, Aba, Sichuan Prov. | 103.228°E | 33.183°N | 3632 | KIZ045304 | H3 (KY172309, KY172492) | A1A1 (KY172587) | A1A1 (KY172623) | A1A2 (KY172536) |
| 20 | *Nanorana pleskei* | China: Hongyuan, Aba, Sichuan Prov. | 103.228°E | 33.183°N | 3632 | KIZ045305 | H3 (KY172310, KY172493) | A1A1 (KY172588) |  |  |
| 20 | *Nanorana pleskei* | China: Hongyuan, Aba, Sichuan Prov. | 103.228°E | 33.183°N | 3632 | KIZ045306 | H3 (KY172311, KY172494) | A1A1 (KY172589) |  |  |
| 20 | *Nanorana pleskei* | China: Hongyuan, Aba, Sichuan Prov. | 103.228°E | 33.183°N | 3632 | KIZ045307 | H3 (KY172312, KY172495) |  |  |  |
| 20 | *Nanorana pleskei* | China: Hongyuan, Aba, Sichuan Prov. | 103.228°E | 33.183°N | 3632 | KIZ-YPX32846 | H3 (KY172313, KY172496) |  |  |  |
| 20 | *Nanorana pleskei* | China: Hongyuan, Aba, Sichuan Prov. | 103.228°E | 33.183°N | 3632 | KIZ-YPX32847 | H3 (KY172314, KY172497) |  |  |  |
| 20 | *Nanorana pleskei* | China: Hongyuan, Aba, Sichuan Prov. | 103.228°E | 33.183°N | 3632 | KIZ-YPX32849 | H3 (KY172315, KY172498) |  |  |  |
| 20 | *Nanorana pleskei* | China: Hongyuan, Aba, Sichuan Prov. | 103.228°E | 33.183°N | 3632 | KIZ-YPX32850 | H3 (KY172316, KY172499) |  |  |  |
| 20 | *Nanorana pleskei* | China: Hongyuan, Aba, Sichuan Prov. | 103.228°E | 33.183°N | 3632 | KIZ-YPX32851 | H3 (KY172317, KY172500) |  |  |  |
| 20 | *Nanorana pleskei* | China: Hongyuan, Aba, Sichuan Prov. | 103.228°E | 33.183°N | 3632 | KIZ-YPX32855 | H3 (KY172318, KY172501) |  |  |  |
| 20 | *Nanorana pleskei* | China: Hongyuan, Aba, Sichuan Prov. | 103.228°E | 33.183°N | 3632 | KIZ-YPX32859 | H3 (KY172319, KY172502) |  |  |  |
| 21 | *Nanorana pleskei* | China: Ruoergai, Aba, Sichuan Prov. | 102.947°E | 33.649°N | 3498 | KIZ045372 | H2 (KY172320, KY172503) |  |  |  |
| 21 | *Nanorana pleskei* | China: Ruoergai, Aba, Sichuan Prov. | 102.947°E | 33.649°N | 3498 | KIZ045374 | H2 (KY172321, KY172504) |  |  |  |
| 21 | *Nanorana pleskei* | China: Ruoergai, Aba, Sichuan Prov. | 102.947°E | 33.649°N | 3498 | KIZ045383 | H2 (KY172322, KY172505) | A1A1 (KY172590) |  |  |
| 21 | *Nanorana pleskei* | China: Ruoergai, Aba, Sichuan Prov. | 102.947°E | 33.649°N | 3498 | KIZ045387 | H3 (KY172323, KY172506) | A1A1 (KY172591) | A1A2 (KY172624) | A1A2 (KY172537) |
| 21 | *Nanorana pleskei* | China: Ruoergai, Aba, Sichuan Prov. | 102.947°E | 33.649°N | 3498 | KIZ045389 | H3 (KY172324, KY172507) | A1A1 (KY172592) |  |  |
| outgroup |  |  |  |  |  |  |  |  |  |  |
|  | *Nanorana parkeri* | China: Longzi, Shannan, Tibetan Autonomous Region | 92.411°E | 28.420°N | 3908 | KIZ-YPX14899 | (KY172325, KY172508) |  |  |  |
|  | *Nanorana parkeri* | China: Nierixiong, Rikaze, Tibetan Autonomous Region | 88.842°E | 29.321°N | 3843 | KIZ-YPX14646 | (KY172326, KY172509) |  |  |  |

Table S2: Primers used in this study.

| Locus | primer name | Primer sequence | Source |
| --- | --- | --- | --- |
| *Cytb* | Cytbs | TAAATCTCACCCCCTCCTCAA | Zhou *et al*. 2012 |
|  | Cytba | AAGAAGATTTTGGCGATGGG | Zhou *et al*. 2012 |
| *COI* | Chmf4 | TYTCWACWAAYCAYAAAGAYATCGG | Che *et al*. 2012 |
|  | Chmr4 | ACYTCRGGRTGRCCRAARAATCA | Che *et al*. 2012 |
| *Tyr* | Tyr1D | TCCTCCGTGGGCACCCARTTCCC | Bossuyt and Milinkovitch, 2000 |
|  | Tyr1F | TCATCTCCCGYCAYCTTCTGGAT | Bossuyt and Milinkovitch, 2000 |
|  | Tyr1G | TGCTGGGCRTCTCTCCARTCCCA | Bossuyt and Milinkovitch, 2000 |
|  | Tyr1B | AGGTCCTCYTRAGGAAGGAATG | Bossuyt and Milinkovitch, 2000 |
| *RAG1* | L-RAG1Ran | CTGGTCGTCAGATCTTTCAGC | Stuart 2008 |
|  | H-RAG1Ran | GCAAAACGTTGAGAGTGATAAC | Stuart 2008 |
| *GCG* | AF-f1 | TCACTCACAAGGGACATTTA | Zhou *et al*. 2012 |
|  | AF-r1 | TCCAAGAACTGGGTTACATC | Zhou *et al*. 2012 |
|  | AF-nr | TTGGGTCTTAATGGAACTGG | Yan *et al*. 2013 |
|  | AF-nf | TAATTGACCAGGGTTTGG | Yan *et al*. 2013 |

**Fig. S1** Population divisions based on rivers for selecting dispersal models. Six regions, A to F, were defined based on rivers. Region A includes localities 5 and 6; region B includes localities 12 and 13; region C includes localities 7 to 11; region D includes localities 2 and 3; region E includes localities 1 and 14 to 21; region F includes locality 4. Pink line indicates Yellow River, green denotes the Dadu River, blue shows Yalong River, and yellow specifies the Jinsha River.Red triangles indicate from east to west the Qionglai, Daxueshan, Shaluli and Mangkang mountains. The semitransparent picture is the reported distribution of *Nanorana pleskei*.


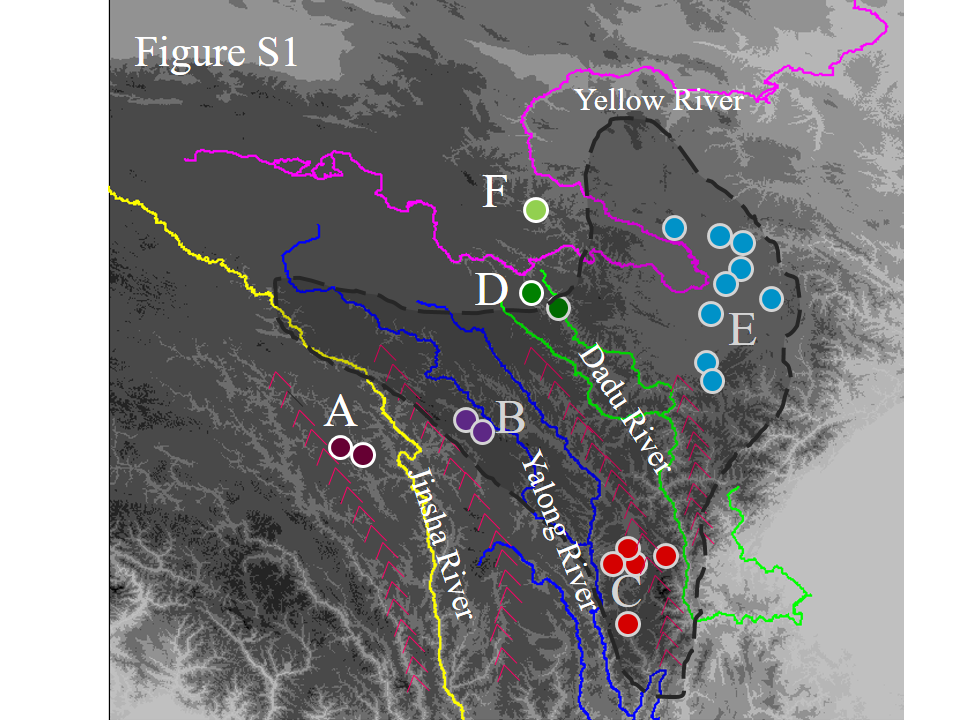


**Fig. S2** Maternal genealogy of *Nanorana pleskei* based on Bayesian inference. Bootstrap proportions (ML and MP trees) ≥70% and Bayesian posterior probabilities ≥95% were treated as strong support (▼). Bootstrap proportions ≥50% and Bayesian posterior probabilities ≥75% were treated as weakly support (▽). Bootstrap proportions <50% and Bayesian posterior probabilities <75% were treated as no support (※).


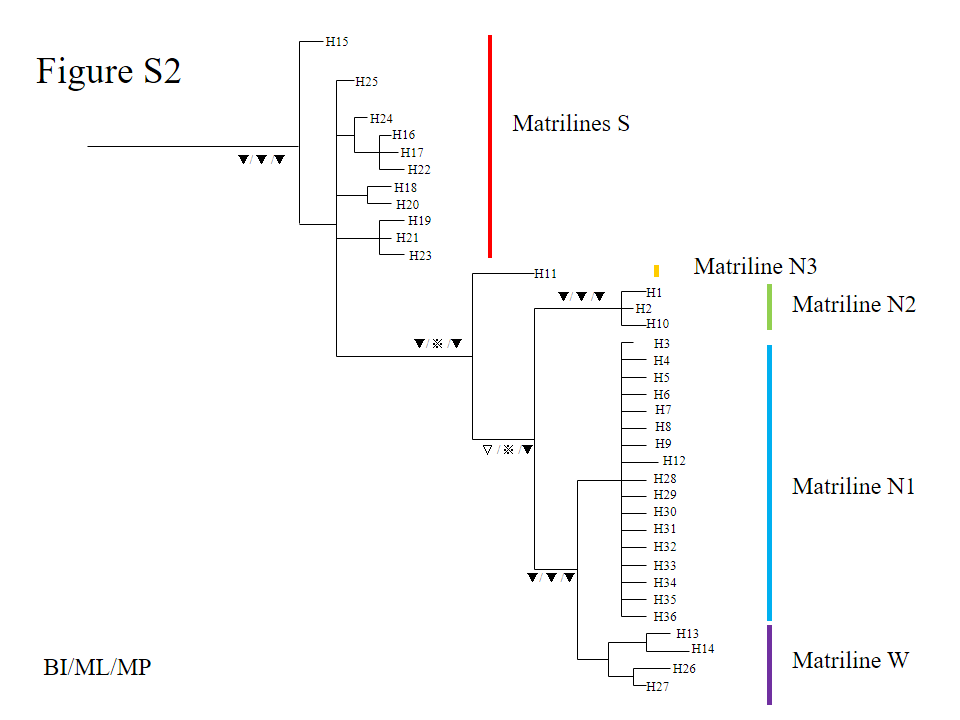


**Fig. S3** Population trees obtained by BEAST. a: consensus tree based on the combined mtDNA and nuDNA data; b: consensus tree based on nuDNA data.


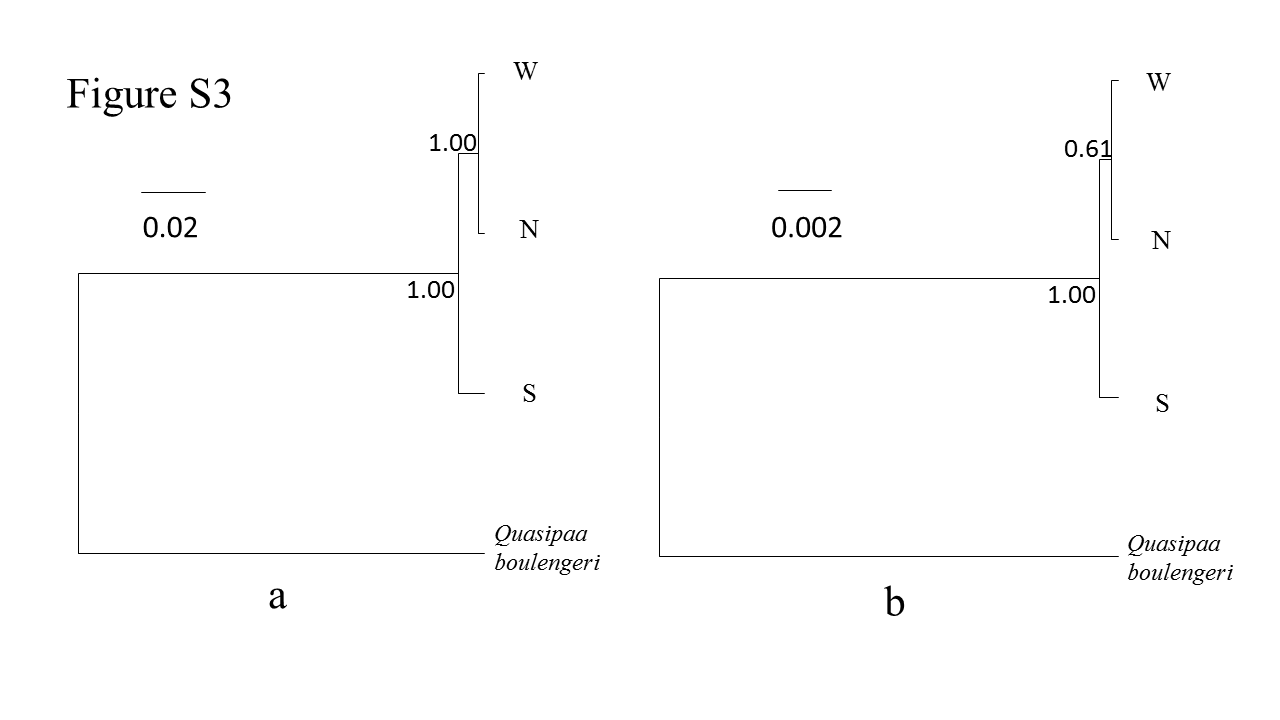


**Fig. S4** The median-joining network haplotypes from *Nanorana pleskei*. Colors correspond to matrilines in Figure 1.


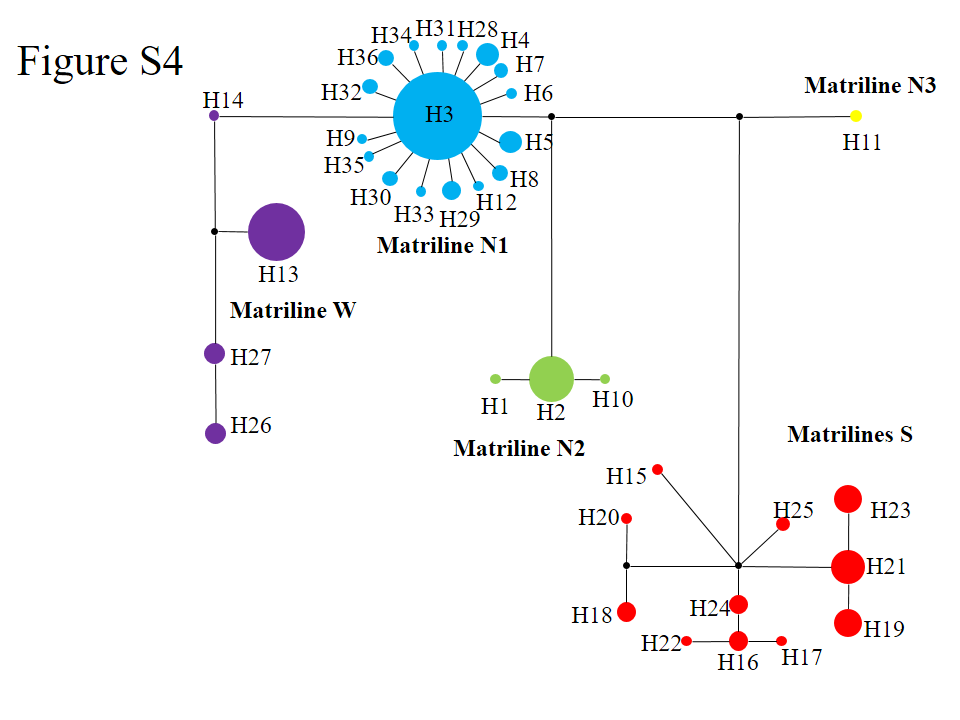


**Fig. S5** Results of SAMOVA. a: K = 5; b: K = 6; c: K = 7.


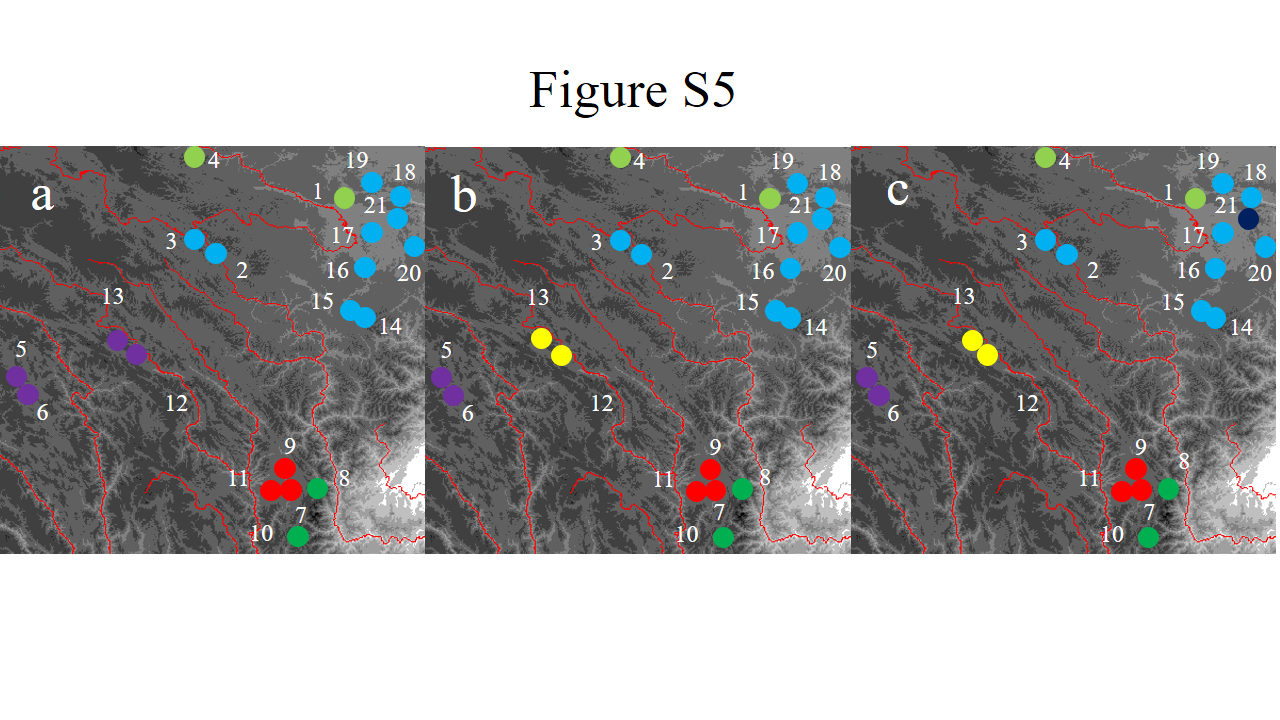

Supplement: Supplementary file 1 [file ECE3-7-240-s001.docx]
